# Refining the diagnostic accuracy of Parkinsonian disorders using metaphenomic annotation of the clinicopathological literature

**DOI:** 10.1038/s41531-025-01157-y

**Published:** 2025-11-10

**Authors:** Quin Massey, Leonidas Nihoyannopoulos, Peter Zeidman, Thomas Warner, Kailash Bhatia, Sonia Gandhi, Christian Lambert

**Affiliations:** 1 Department of Imaging Neuroscience, UCL Queen Square Institute of Neurology, London, UK; 2 Queen Square Brain Bank for Neurological Disorders, UCL Queen Square Institute of Neurology, London, UK; 3 Department of Neurodegenerative Disease, UCL Queen Square Institute of Neurology, University College London, London, UK; 4 Department of Clinical and Movement Neurosciences, UCL Queen Square Institute of Neurology, London, UK; 5 The Francis Crick Institute, London, UK

**Keywords:** Parkinson's disease, Predictive medicine

## Abstract

The diagnostic precision of Parkinsonian disorders is not accurate enough. Even in expert clinics, up to one in five diagnoses are incorrect. Gold standard diagnosis is post-mortem confirmation of the underlying proteinopathy; however, many clinicopathological studies focus on either a single disease or frame analyses in one temporal direction that may underestimate the true extent of mis- and missed diagnoses. We identified 125 published clinicopathological studies since 1992, extracted phenotype information for ~9200 post-mortem cases, curated the data in a standardised machine-readable format and used this to develop a probabilistic model to quantify diagnostic likelihood based on clinical observations. We found diagnostic accuracy was highest for multiple system atrophy (MSA, 92.8%) and lowest for dementia with Lewy bodies (DLB, 82.1%). MSA and progressive supranuclear palsy were most frequently mis-labelled as Parkinson’s disease (PD) in life (7.2% and 8.3% of cases), whereas the most common PD misdiagnosis was Alzheimer’s (~7% cases). We calculated likelihood ratios for a large range of clinical phenotypes and demonstrated how these can be used to help refine and improve diagnostic accuracy. This work delivers a harmonised, open-source dataset representing over 30 years of published results and represents a key foundation for flexible predictive models that leverage different sources of information to better discriminate Parkinsonian disorders during the early and prodromal phases of the illness.

## Introduction

Parkinson’s disease (PD) is the second most common neurodegenerative illness. It presents as a motor syndrome that emerges once 60%–70% of the nigral dopaminergic neurons have been irreversibly lost^1^. Diagnosis is clinical, based on the cardinal signs of bradykinesia with rigidity and/or tremor, coupled with a lack of features to indicate an atypical Parkinsonian syndrome (aPD)^1^. Once diagnosed, progression is highly variable, with survival ranging from a few years to several decades^2^.

The diagnostic precision of Parkinsonian disorders is not accurate enough. Even in expert clinics, up to one in five PD diagnoses are incorrect^3^. aPD conditions are common mimics, which include Multiple System Atrophy (MSA), Progressive Supranuclear Palsy (PSP), dementia with Lewy bodies (DLB) and Corticobasal Degeneration (CBD)^3^. Whilst several data-driven methods allow identification of possible subtypes in life^4^, to date these do not show differences at a clinicopathological level and, in fact, approximately 50% of the more aggressive forms of PD, the so-called *malignant* phenotype identified data-driven methods, are misdiagnosed as aPD in life meaning across all of the Parkinsonian conditions the most extreme forms of progression (both fast and slow) are likely misclassified^5^. These represent a challenge for developing disease-modifying treatments, as clinical trial cohorts will contain mixtures of pathologies (*misdiagnoses*) necessitating larger sample sizes to detect a signal, and subtypes of disease with markedly different disease trajectories, that may require more aggressive or targeted therapies^6^, will be under-represented (*missed diagnoses*).

The diagnostic gold standard for these disorders is post-mortem confirmation of the underlying proteinopathy. However, many clinicopathological studies focus on either a single diagnostic entity or frame the analyses in one temporal direction (i.e., life diagnosis vs post-mortem findings or vice versa). Given that each of the Parkinsonian disorders can mimic one another, these risk missing the true extent of mis- and missed diagnoses. The value of pathologically confirmed cases is high, and there is a wealth of data embedded in historic reports that could be leveraged to help improve diagnostic precision in life.

Phenotypes are defined as *“any observable characteristic of an organism”*^7^ and therefore span many body systems and multiple levels of scale. Often, the terminologies used to define different observations vary between experimenters, making systematic comparisons hard. To tackle this complexity, ontologies seek to formalise and structure the language used to describe different observations, making them more suitable for large-scale computational analyses and comparisons^8^. In human disease, the Human Phenotyping Ontology (HPO) is a highly successful framework for deep phenotyping (https://hpo.jax.org/app/). Whilst there has been an independent Parkinson’s disease ontology (PDON)^9^, HPO is actively maintained, regularly updated through community feedback to iterate and refine, and has been adopted by large initiatives such as the 100,000 Genomes Project.

*Metaphenomic annotation* is a novel method to structure data from published cohorts or single case reports in a standardised, machine-readable format based around internationally recognised phenotyping ontologies and structures, leveraging the Phenopacket standard for structuring phenotype data (http://phenopackets.org). It allows more efficient pooling of phenotyping data that can then be used for a wide array of different analyses.

In this work, we used metaphenomic annotation on the clinicopathological literature for Parkinsonian disorders published since the 1992 validation of the Queen Square Brain Bank Criteria for PD^10^. The objective was to comprehensively map the mis- and missed diagnoses across the main Parkinsonian disorders and link these gold-standard cases to the phenotypic features observed in life. These results form the foundation for a naïve Bayesian classifier^1^, that can be used to quantify the probability of disease for each of the main Parkinsonian syndromes. These results can be flexibly expanded or incorporated into other tools, modalities or risk scores seeking to improve the diagnostic accuracy across the Parkinsonian disorders, and deliver a freely accessible, machine-readable library summarising the last 30 years of published data.

## Results

### Cohort

**Fig. 1:**
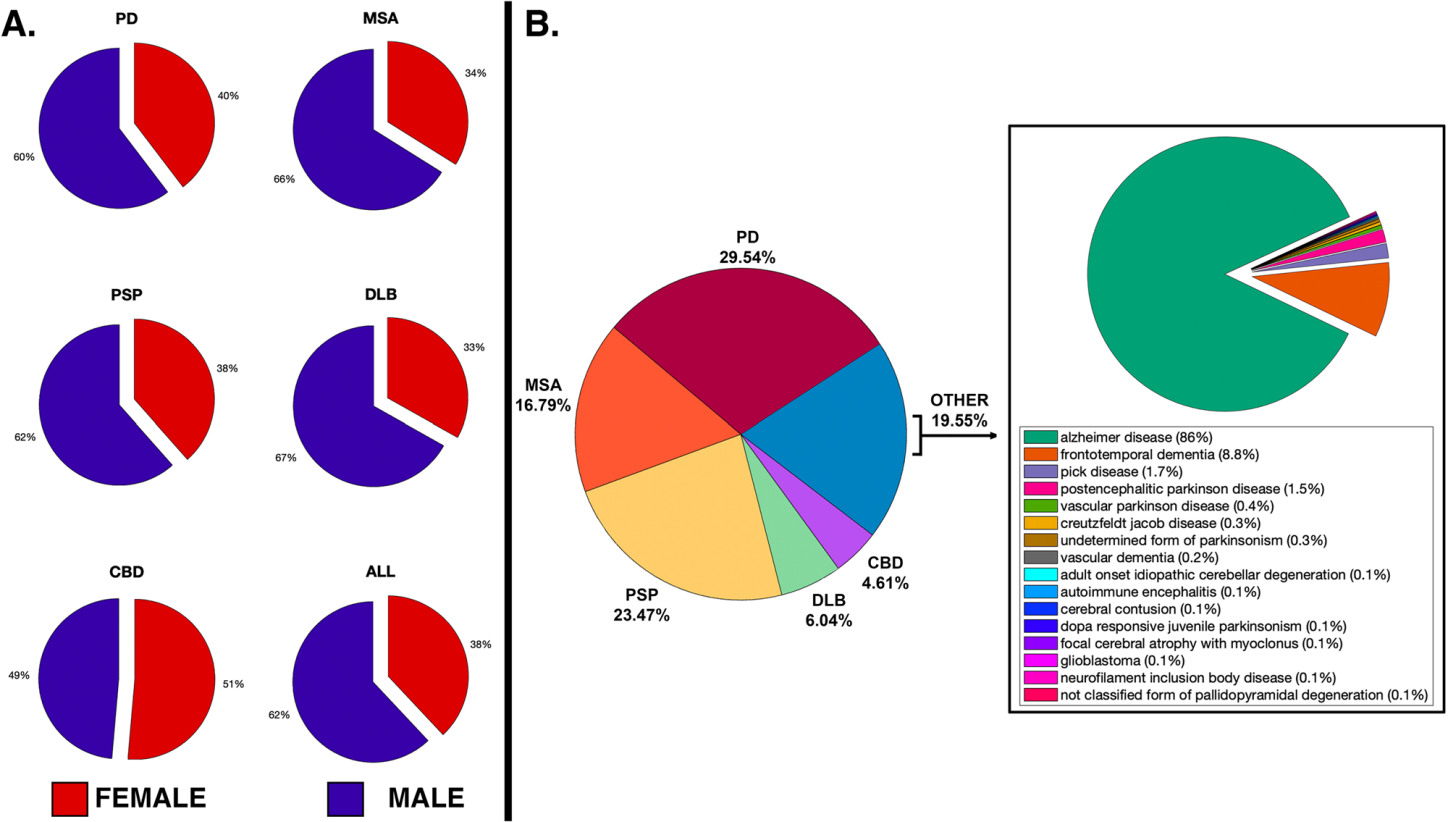
Clinicopathological dataset summary. **A** Summary of female-male ratios; **B** Summary of post-mortem diagnoses.

125 publications were identified, generating 610 annotations totalling 9287 post-mortem diagnosed cases (2406 PD, 1594 MSA, 1835 PSP, 834 DLB, 354 CBD, 2264 other), which were used for age of onset and survival analyses. Of these, only 4341 (46.7%) reported biological sex, with a female:male ratio of 38:68% overall (see Fig. 1A). 5748 cases provided misdiagnosis data (1698 PD, 965 MSA, 1349 PSP, 347 DLB, 265 CBD, 1124 other). The “*other*” diagnostic category was most frequently Alzheimer’s disease (86%), followed by Frontotemporal dementia (8.8%), with the PSP and CBD cases contributing significantly to the latter. This is summarised in Fig. 1B.

### Age of onset

**Fig. 2:**
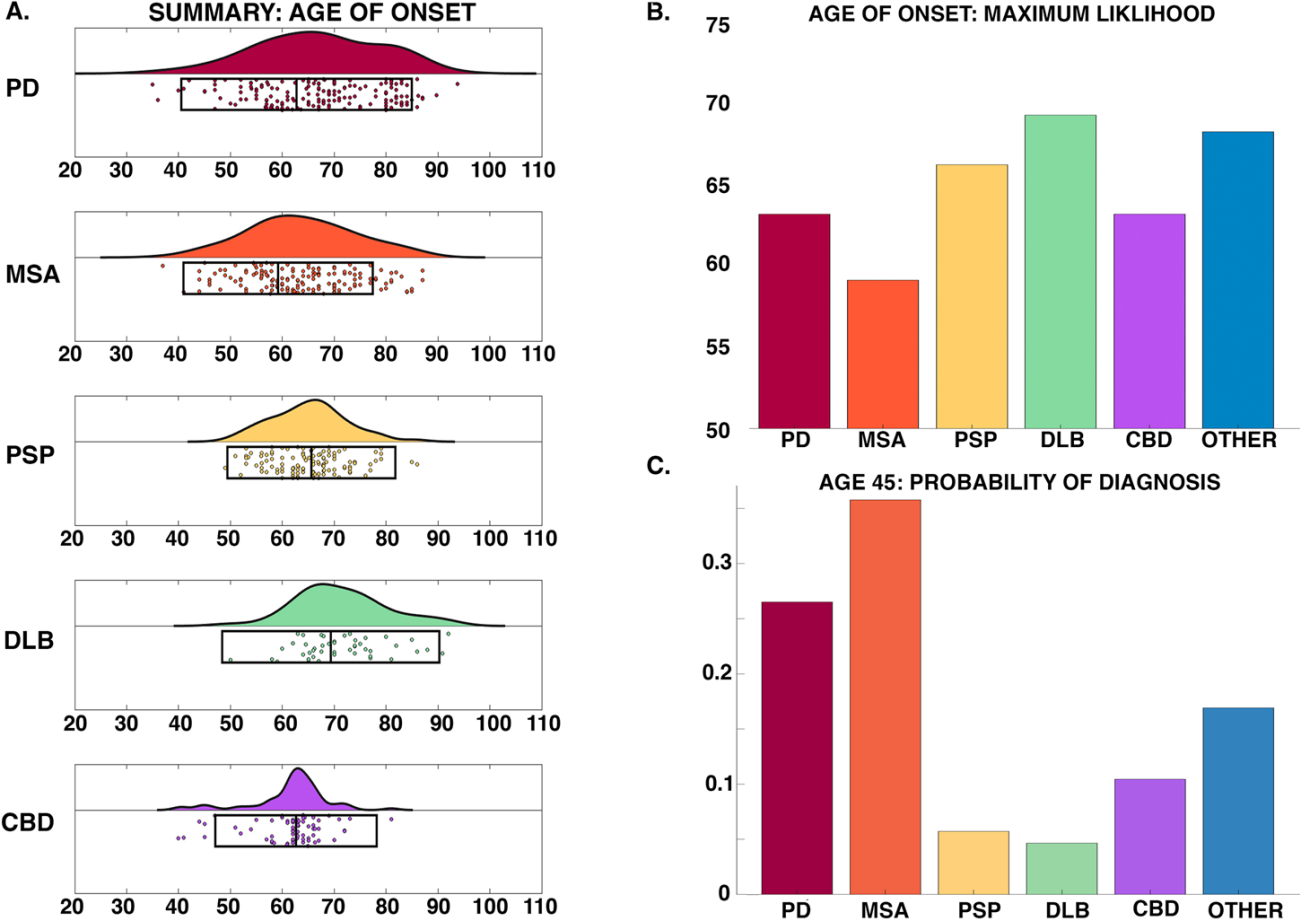
Age of onset. **A** Raincloud plots summarising 9287 Parkinsonian cases – boxes below indicate weighted mean and two standard deviations. Note that each scatter point may either represent a cohort study or single case reports; however, these were weighted by sample size to calculate summary statistics and probabilities. No significant difference in age of onset was seen between groups; **B** Age of onset, maximum likelihood for each condition; **C** Probability of each Parkinsonian syndrome at age 45 y.

**Fig. 3:**
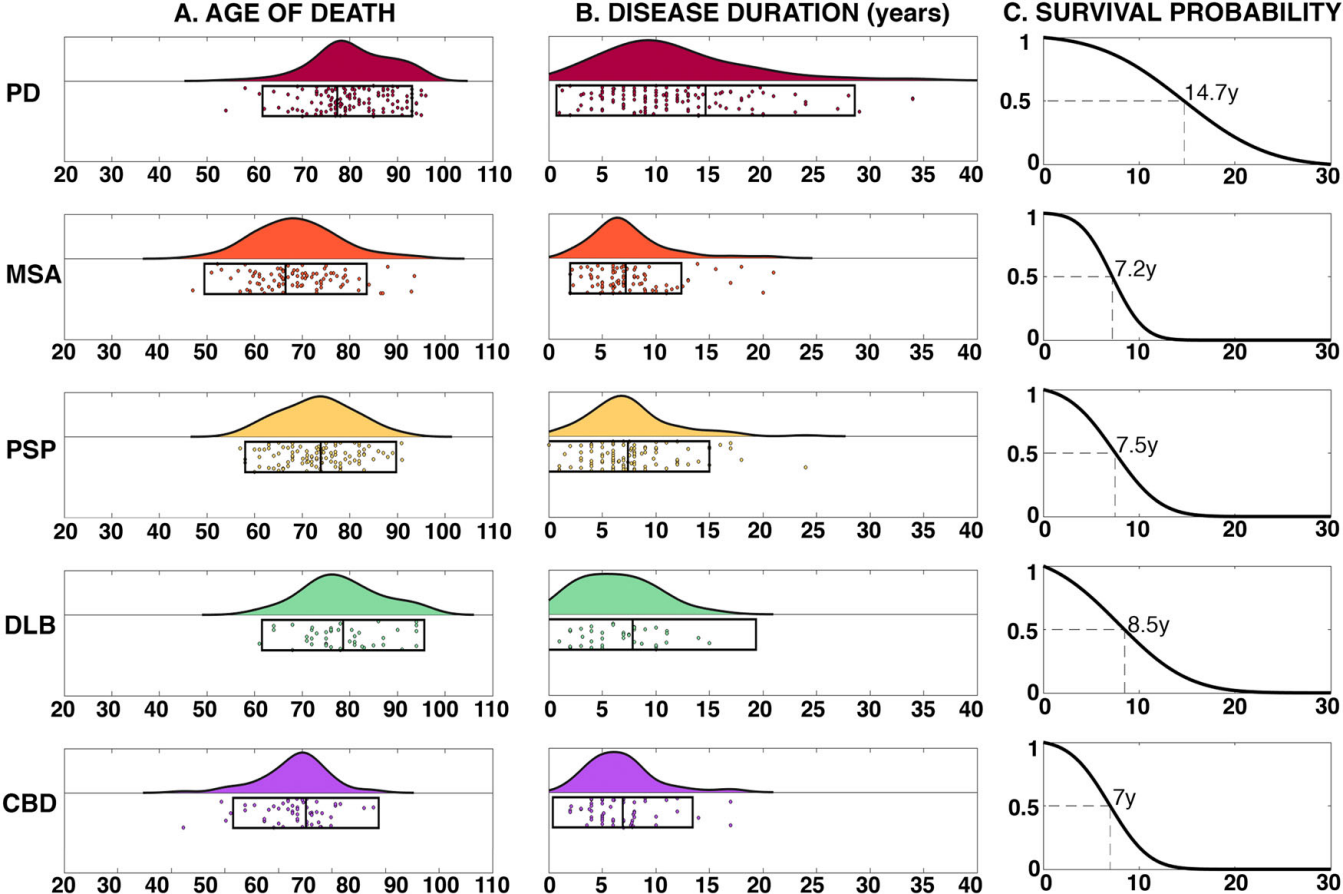
Survival. **A** Raincloud plots summarising age of death for each condition; **B** Raincloud plot summarising disease duration in years; **C** Cumulative probability of survival from symptom onset in years with 50% survival point labelled.

Figure 2 summarises these results. Symptom onset for DLB was the oldest (69.34 ± 10.46 y), followed by PSP (65.60 ± 8.10 y), PD (62.75 ± 11.11 y), CBD (62.64 ± 7.78 y) and MSA the youngest (59.19 ± 9.12). DLB was significantly older than the other groups, except for PD, and CBD was significantly younger, except for MSA (Fig. 3).

### Survival

Figure 3 summarises the mortality data: DLB has the oldest mean age of death (78.59 ± 8.52 y), followed by PD (77.37 ± 7.86 y), PSP (73.87 ± 7.93), CBD (70.77 ± 7.64), and then MSA (66.49 ± 8.52). PD and DLB were significantly older than the rest of the groups, and CBD and MSA were significantly younger than PSP (Fig. 5). Duration of survival for aPD was similar to MSA (7.19 ± 2.60), PSP (7.39 ± 3.80), DLB (7.85 ± 5.75) and CBD (6.91 ± 3.26). PD survived significantly longer (14.64 ± 6.96) with a disease duration ranging from 2 to 34 years.

### Misdiagnosis

**Fig. 4:**
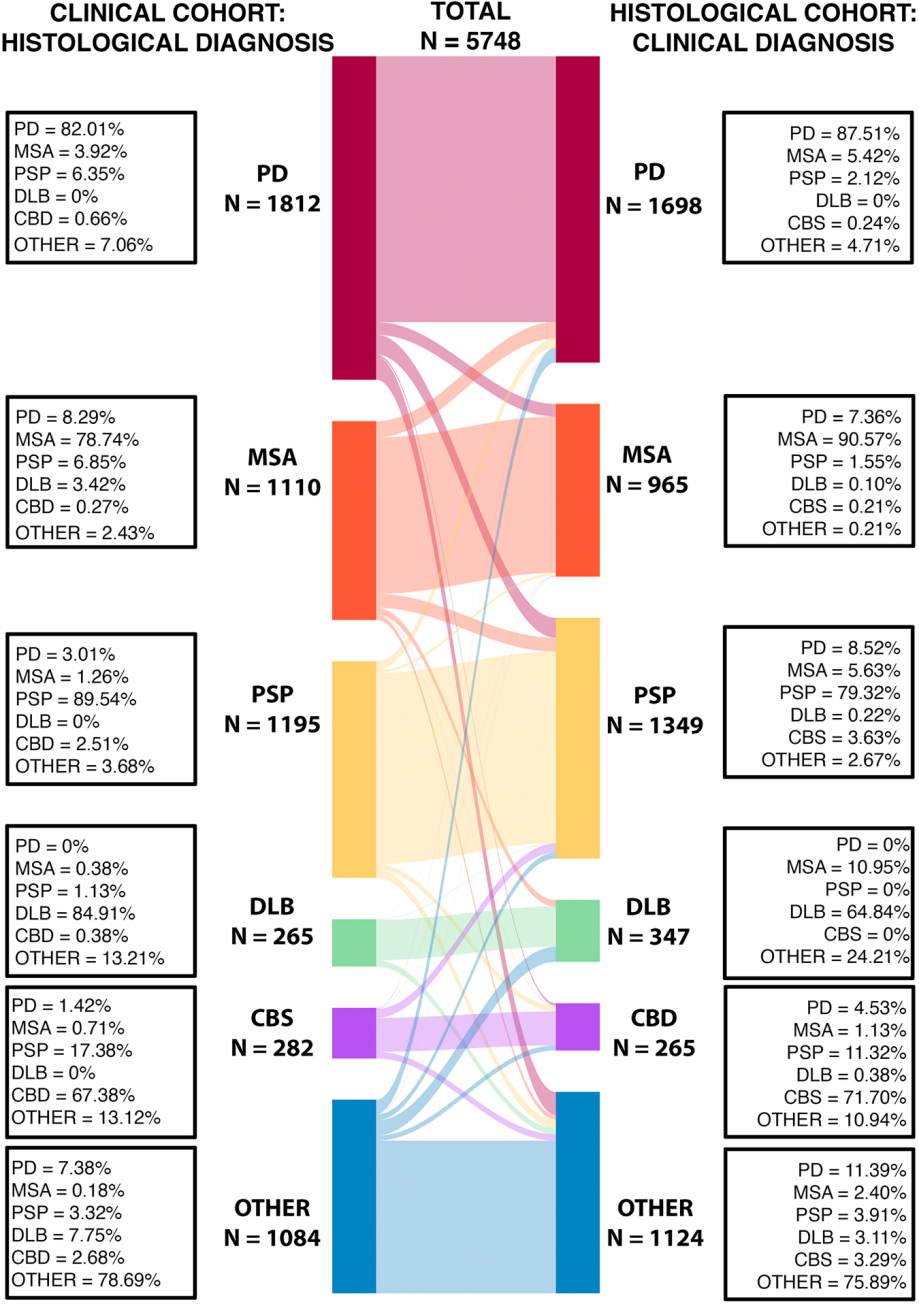
Diagnostic errors. Misdiagnosis (left, clinical diagnosis mapped to post-mortem) and missed diagnosis (right, post-mortem mapped to clinical label) between conditions. Note in life, CBD is categorised as corticobasal syndrome (CBS).

This is summarised in Fig. 4. Balanced accuracy was lowest for DLB, with a significant number labelled as MSA or OTHER (mainly AD). CBD was the next lowest, however, in life this presents as corticobasal syndrome that is often due to PSP or FTD, as reflected in our results (17.38% PSP, 13.12% other). Of note, ~5% of CBD cases are labelled as PD in life. PSP was next due to the lower sensitivity compared to PD and MSA. This was caused by in-life PSP mimics caused predominantly by PD (3.01%), CBD (2.51%) and OTHER (3.68%), and cases of PSP being mislabeled as PD (8.52%), MSA (5.63%) and CBD (3.63%). The balanced accuracy for PD was ~90%, but with comparatively large proportions of AD mimics (~7%) in life and with ~8% of cases misdiagnosed as aPD, most often MSA (5.42%). This latter result is in line with observations that up to half of the more aggressive, malignant forms of PD are diagnosed as aPD^5^. MSA was the most accurate overall with a balanced accuracy of 92.82%, and similar numbers of PD and PSP mimics in life (8.29% and 6.85%, respectively). Between the more common aPD conditions, PSP and MSA, there were similar numbers being mislabeled as PD in life (7.36%, 8.52%). There is evidence in PSP that these Parkinsonian variants follow a less aggressive course with longer survival, but not in MSA^11^. A table summarising the pooled diagnostic accuracies is provided in the Supplementary Data (S2.4).

### Improving diagnostic accuracy using phenotypic data

**Fig. 5:**
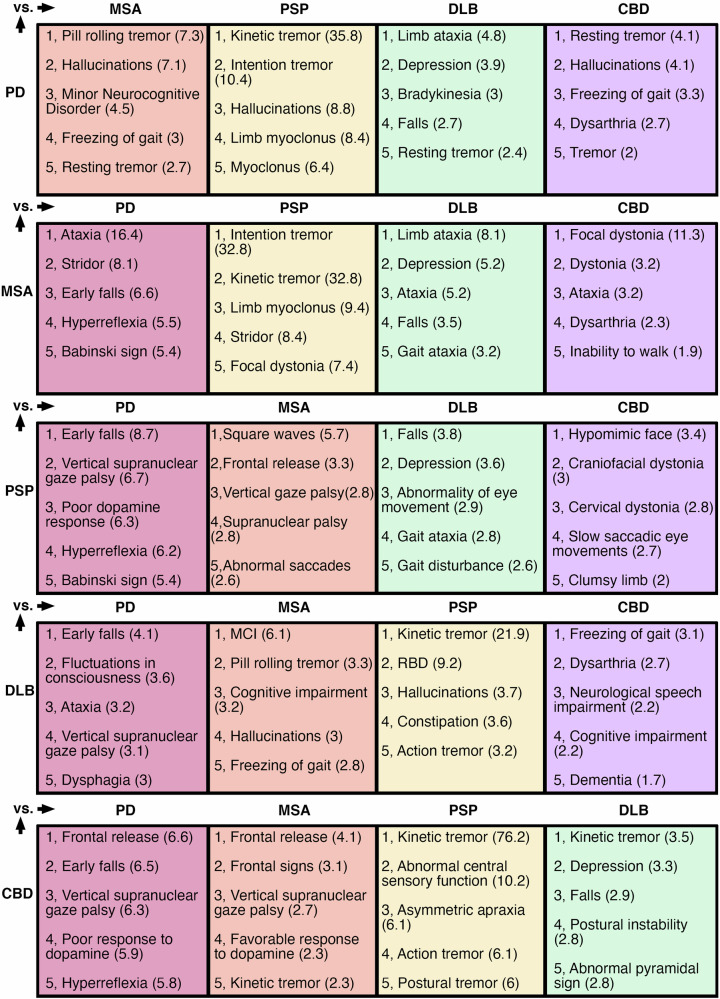
Overlapping clinical signs between Parkinsonian syndromes. Overlapping phenotypes with the maximum likelihood ratio over the entire HPO tree for discriminating the condition on the left from each of the main mimics (top row). Positive likelihood ratio provided in the brackets. MCI mild cognitive impairment. RBD REM sleep behaviour disorder. Most of the terms are as per HPO definitions, but a few were abridged due to space constraints.

88% of studies had extractable phenotyping data, providing 4076 descriptors. These were mapped to 245 unique HPO descriptors over 12 parent domains (Supplementary Data S4). From this HPO graph, we calculated the top five phenotype terms with the largest likelihood ratios between diseases, reflecting clinical observations that can co-occur in both diseases and best discriminate between the two (Fig. 5). This unbiased approach confirms certain highly predictive clinical features such as pill-rolling/rest tremors in PD, ataxia and stridor in MSA, cognitive impairment DLB, gaze palsies and falls PSP, and cortical sensory loss CBD (Fig. 5). This analysis excluded empty observations, which may pathognomonic signs for one disease versus another, because we could not assume absent reporting equated to an absent sign. Relaxing this constraint confirms this assumption. For example, cortical sensory loss has never been reported in PD, MSA and DLB, alien hand phenomena in MSA or DLB, and pill-rolling tremor in PSP, but then the inability to walk has not been recorded in DLB, which is clearly an artefact.

Despite the limitations of incomplete reporting in post-mortem literature, providing a robust link between phenotype and pathological diagnosis provides a foundation that can be developed to improve diagnostic accuracy in vivo, as shown in the following example:

A 50-year-old person presents with Parkinsonism, REM Behaviour Sleep Disorder (RBD), orthostatic hypotension^5^ and tremor. Leveraging the metaphenomic structured data, we can calculate that the most likely diagnosis is PD (probability = 0.97) or DLB (0.73), followed by MSA (0.58). The strength of this approach is that it can easily incorporate new data to refine the prediction. If subsequent testing revealed an elevated neurofilament light chain level^12^, updating the calculation with the corresponding likelihood ratios would result in probabilities of 0.89 for MSA and 0.83 for PD (Table 1). If, on re-examination, a rest tremor was found, then PD would remain the most likely even with elevated NFLC (PD 0.99 vs MSA 0.78), but an additional history of erectile dysfunction would then make MSA more likely (MSA 0.99 vs PD 0.88).

**Table 1:** Probabilsitic diagnostics worked example

Contrast	Tremor	Orthostatic hypotension	Rapid eye movement sleep behaviour disorder	TOTAL LR	Pre-test probability	Post-test odds	Probability	Probability with elevated NFLC
**PD vs MSA**	2.23	1.25	0.85	92.49	0.32	29.19	0.97	0.83
**PD vs PSP**	3.32	2.23	4					
**PD vs DLB**	2.32	1.3	0.44					
**MSA vs PD**	0.45	0.8	1.18	3	0.45	1.36	0.58	0.89
**MSA vs PSP**	1.49	1.79	4.73					
**MSA vs DLB**	1.04	1.05	0.52					
**PSP vs PD**	0.3	0.45	0.25	0	0.14	0	0	0
**PSP vs MSA**	0.67	0.56	0.21					
**PSP vs DLB**	0.7	0.58	0.11					
**DLB vs PD**	0.43	0.77	2.29	30.34	0.09	2.76	0.73	0.31
**DLB vs MSA**	0.96	0.96	1.94					
**DLB vs PSP**	1.43	1.71	9.17					

Worked example of a 50yo with Parkinsonism plus RBD, Orthostatic Hypotension and tremor. The most likely diagnosis with this combination is PD (0.97) or DLB (0.73), followed by MSA (0.58). However, if this individual has an elevated NFLC result, this flips, making a diagnosis of MSA more likely (0.89 vs 0.83). Ascertaining additional clinical signs enables one to refine this further – Building on the example above, if a rest tremor was present, PD would remain the most likely even with elevated NFLC (PD 0.99 vs MSA 0.78), however the additional presence of erectile dysfunction with the rest tremor then makes MSA far more likely (MSA 0.99 vs PD 0.88). CBD not shown as RBD has not been reported in this condition.

## Discussion

This work structures 30 years of clinic-pathological literature for the main Parkinsonian syndromes into an easy-to-use, machine-readable format for complex phenomic data. It establishes a foundation for clinical observations within a probabilistic diagnostic framework that underwrites transportability by design by allowing a “*plug and play*” approach where different combinations of techniques can be used and adapted to local resources. Importantly, such an approach provides quantitative metrics that are directly comparable irrespective of the combination of methods used and implicitly accounts for the underlying uncertainty inherent in all in vivo diagnostics.

Onset ages were in keeping with existing literature^13^, with MSA more likely at a younger age versus PSP and DLB, which tended to be later. The range was substantial, particularly in PD and MSA, where it spans nearly six decades (35 y–94 y). Survival in aPD was approximately 7 years, with no differences between groups. This falls within the expected range for PSP and MSA^14^, but for DLB was at the upper end of the expected 1.9–6.3 y^15^. This discrepancy may emerge for several reasons: DLB can be challenging to disambiguate from AD in life^16^, as hallucinations and Parkinsonism may occur in both^17^, and at post-mortem, a significant proportion have dual pathology associated with more rapid progression and shorter survival^18^. Furthermore, DLB and PD dementia look identical at post-mortem, and the distinction hinges upon the sequence and timing of clinical events. As such, these cases are relatively under-represented in the literature (Fig. 1), and the overlap with AD may bias current cohorts. PD was associated with a longer survival (14.64 ± 6.96 y), consistent with previous reports^19^, ranging from 1 to 34 years. The reasons for this heterogeneity are unknown, with age of onset, akinetic phenotype, cognitive dysfunction and GBA1 gene variants all associated with more rapid disease progression^19^, whereas lifestyle factors such as physical exercise seem to exert protective effects.

The diagnostic accuracy of Parkinsonian disorders remains suboptimal, and is known to be partly dependent on clinician experience, years from symptom onset and clinical phenotype^13^. For PD, balanced accuracy was 89.7%, in line with Adler and colleagues^20^. For MSA, it was >90%, higher than the expected 70%–80%^21^. Reasons for this may include taking the final diagnosis at death, a greater sample size and improvements in the diagnostic criteria over time^22^. Accuracy for DLB was one of the lowest, with many cases mislabelled either as AD or MSA. As noted, AD co-pathology is common in DLB, which may make the distinction tricky. The presence of autonomic failure is likely to account for the confusion with MSA. Whilst cognitive impairment was considered atypical for MSA, it has become increasingly recognised^23^, although it occurs later in the disease. In line with previous work, AD was the most frequent “*other*” diagnosis across all conditions, particularly LBD. Parkinsonism is common in AD^24^, with similar dopaminergic cell loss associated with regional neurofibrillary tangles^24^.

There have been many attempts to use data-driven methods to identify subtypes of clinical progression in life. These have resulted in a multitude of different proposed clusters that tend to largely parse extremely fast progressing forms (e.g., malignant Parkinson’s), intermediate, and extremely slow (benign). Whilst some of these are reproducible across datasets and seem to inform a likely trajectory in life^4^, to date, these have shown no differences at post-mortem^5^. Furthermore, one of the key limitations across all these data-driven methods is that the most extreme forms of each condition will invariably be missed, e.g., 50% of malignant Parkinson’s, extreme fast progressors, are misdiagnosed as aPD in life, 25% of essential tremor syndrome cases have Lewy Body pathology at post-mortem that likely represent extreme slow progressors^25^. Whilst invariably this reflects clinician bias, based on their a priori expectation of how certain conditions should behave, it does mean the most extreme phenotypes are being missed by focusing on one condition alone. This represents an ongoing open challenge for the field, and it is likely that future methods will need to start factoring these considerations into their experimental design. Probabilistic approaches that can leverage multiple emergent diagnostic tools (e.g., SAA, metabolomic profiles, imaging-based classifiers, etc.) provide a simple yet powerful way of objectively weighting these likelihoods across traditional, clinician-based diagnostic boundaries and represent one way to try and start addressing this challenge.

The prodromal phase of Parkinsonian disorders ranges between 5 and 20 years^1,26^. Identifying individuals during this period is critical for disease-modifying therapies. However, a key factor is how accurately can we identify these conditions and discriminate them from potential mimics? Given the substantial heterogeneity within clinic-pathological defined cohorts, this remains a major challenge. It is unlikely that there will be one “*best test*” that will work across all disorders, be universally available and feasible in all scenarios. More likely, a tactical combination of investigations combined with clinical knowledge will need to be applied at the individual subject level^27^, and there will be a trade-off between how much new information each test provides, how invasive the procedure is, patient choice, availability and cost. For example, idiopathic anosmia is a risk factor for PD, with 1 in 10 individuals later developing the condition. There is a new CSF RT-Quic test to detect abnormal alpha synuclein aggregation with a sensitivity of 98% and specificity of 95.3^28^. Assume we want to use this to diagnose pre-motor LBD. If 10,000 anosmics are tested, 1000 will have early LBD, and this test will detect 980 of them (sensitivity 98%). However, 9000 will not have LBD but 432 individuals will have a positive test (i.e., specificity of 95.3), meaning ~30% of the positive diagnostic tests in this scenario will not have LBD.

The probabilistic approach to diagnosis and stratification offers a powerful framework to flexibly combine different techniques and boost diagnostic accuracy, without having to commit to one single approach, test or method, thereby providing something more universally applicable across different healthcare systems and scenarios (for example, via a website or offline app). If the same example of anosmia is viewed in terms of probabilities (here, the pre-test probability is 0.10, and LR+ for RT-Quic is 20.41), a positive test alone equates to a 0.67 probability of prodromal LBD. Viewed in this light, a higher degree of confidence would be warranted before committing to a diagnosis and lifelong treatment or recruitment to a clinical trial. This can be achieved through some simple additional details^27,29^. If we add the stipulation that they are all older than 60, the probability increases to 0.91, with additional features such as subtle motor abnormalities boosting it to 0.99 (vs <0.001 if anosmic over 60 where these other features are absent)^29^. This example is summarised in Supplementary Fig. S3.

This process can also be used in reverse, to identify the most informative tests, investigations or clinical findings that would best help reconcile a diagnostic dilemma and provide an upper bound on the degree of confidence that can be achieved via different approaches. This functionality will be of particular use in more limited healthcare settings, as the clinician can then select the most informative tests available to them and potentially achieve similar levels of diagnostic accuracy through a tactical combination of clinical observations and more widely available tests. Finally, it could be used to refine diagnostic criteria, which all currently rely on a step-wise, categorical approach to try and achieve the right balance between sensitivity and specificity, but invariably results in missed cases. The diagnostic guidelines for CBD provide a good example^30^: Rest tremor is currently an absolute exclusion, but in this work was present in 14% of post-mortem cases that assessed tremor (*N* = 132), and ~5% CBD cases were mislabeled as PD in life. Viewing this same information as probabilities reveals the likelihood of CBD with rest tremor at the age of 50 is ~2%, compared to ~96% PD, which could then be modified by the presence/absence of other clinical features or biomarkers.

The idea of using of using likelihood ratios coupled with Bayesian methods to calculate diagnostic probabilities is not new – It was first described in the seminal 1959 Science publication “*Reasoning Foundations of Medical Diagnosis*”^31^ by Ledley and Lusted, but the uptake of these methods has waxed and waned over the interceding years. Part of the barrier likely relates to generating and aggregating the necessary data to do this well at scale, access to the necessary compute to implement the methods and making the results easy to interpret and use in a clinical setting. The MDS Research Criteria for Prodromal Parkinson’s Disease^1^ is an excellent example of how these methods can be applied to Parkinson’s and served as an inspiration for this work. The key distinction is that the original MDS approach is framed around a relatively narrow binary problem space, asking “*what is the likelihood this is Parkinson’s or not*” in the context of the prodromal period. In contrast, here we have designed and built a complementary approach that is able to ask a more naturalistic many-to-one question “*What is the likelihood this is a Parkinsonian disorder, and of those which is the most likely?*” that can be applied at any point in the illness, both prodromal and manifest (Supplementary Fig. 8). Furthermore, we have delivered the largest, most comprehensive standardised dataset that allows likelihood ratio estimates to be calculated for hundreds of clinical signs across each of the main conditions, all of which clinicopathologically confirmed. Finally, we have shown that once the data is available, inverting these models provides a powerful way to identify what new observation(s) would best discriminate a particular differential diagnosis.

There are several limitations with this current work. Whilst every effort was made to review and annotate all available literature, some could not be obtained or were not in English. However, given the overall numbers, we do not feel this will significantly impact the result. Whilst none of the annotated publications included clearly identical data duplicates, some individual cases may have been duplicated in aggregated large cohort studies. Whilst there is a risk these may weight certain summary statistics (e.g., age, disease duration), given the overall numbers available for analysis, the impact of this will be minimal. Furthermore, our primary motivation for being more inclusive, despite the small risk of duplicate cases, was to ensure we had the broadest possible coverage of the phenotype landscape, and each publication used represented a unique combination of cohort and reported features. Another limitation relates to the changes in diagnostic criteria that have happened over time for each condition, which may influence clinical diagnostic accuracy. It is not possible to integrate this directly into the analysis, as the precise criteria used for each case were often not reported, and clinicopathological cohorts often extend back many years, crossing changes in criteria. As an additional step to assess this, we also checked for changes in diagnostic balanced accuracy over time (Supplementary Data S2.5) – For PD, MSA and PSP, this did not show any clear change, however prior to 2008 the number of published DLB papers were very low biasing towards high accuracies, but that has fallen since greater numbers have started to be reported. CBD shows a significant drop after 2012, which may reflect an update in the clinicopathological diagnostic guidelines coupled with a drop in the number of published cases. Despite this, the pooled accuracies reported in Fig. 4 and Supplementary Data S2.4 are likely to provide a reasonable estimate of diagnostic accuracy simply through the law of large numbers, which is supported by our results being largely in line with previous reports. Furthermore, the focus of our work was on extracting objective measures, such as age and phenotype, based on clinicopathologically confirmed cases to allow us to calculate relative probabilities, which are unlikely to be significantly impacted by these issues. Furthermore, certain cohorts were difficult to classify using the initial framework, specifically those with dual diagnoses. Whilst in the annotation, we included a separate *“dual diagnosis”* category if this was clearly identifiable and extracted histological staging data, the distinction between two diseases versus low-level mixed proteinopathies is not well defined nor historically reported, and represents an open challenge. There was marked heterogeneity in precisely what was reported in the literature in terms of diagnostic milestones, demographics and phenotypic features. Regarding the latter, it had to be assumed that a lack of reporting did not equate to absent signs, which had the problem of conflating publication bias for rarer features. We attempted to factor for this by imposing a minimum number of observations, but moving forward, another option would be to repeat this approach with clinical data from large cohort studies in life, where we can quantify the probability of misdiagnosis using the data collected here to better combine the two. This deeper coverage may also allow the inclusion of “*pathognomonic”* phenotypes (i.e., those with an infinite LR), which we excluded from this work due to the patchy and inconsistent reporting in published reports. Whilst we had originally wanted to integrate temporal information (i.e., when certain clinical features emerge), this was relatively rare in published studies outside single case reports. Consequently, we treated each feature as the *likelihood of observing a phenotype at any point in the disease,* so we could aggregate all observations to more reliably estimate probabilities. Admitted, this will be a less precise estimate than one that could properly incorporate temporal information, but it is sufficient to provide a first pass approximation of the proposed framework, as it holds equally true for all diagnostic entities being considered. Supplementary Fig. 8 shows a worked example for a clinicopathological MSA case report published in NEJM (Case 27-2004) detailing from prodrome to death that demonstrates this. Finally, one limitation of naïve Bayesian classifiers is the assumption that the observations used are conditionally independent. However, as highlighted by other authors^1^, it is impossible to determine whether many clinically based markers are truly independent. To account for this, we would recommend a pragmatic approach by selecting observations that can be considered reasonably independent, particularly when viewed from an anatomical/circuit level perspective (i.e., asking if there could there be a direct causal link between the mechanism that causes one phenotypic feature and another). Provided one avoids using multiple terms that are obviously highly collinear and would disproportionately bias one feature-domain much more than others, for example, including rest, postural and action tremor all together, any bias introduced by weakly collinear terms would largely cancel out by normalising the probabilities over multiple conditions. Additionally, the HPO hierarchy provides a principled way to help select reasonably independent observations and deal with obviously collinear terms, by aggregating these into phenotypes from higher up the hierarchy (e.g., subsuming multiple tremors into the single HPO term “tremor” for the preceding example).

Whilst this work highlights the power of leveraging existing, large cohorts and data to help develop new tools to refine and improve diagnostic accuracy, it also reveals the stark limitations caused by the lack of standardisation across disciplines, specialists and journals for reporting and describing neurological cohorts. Establishing a commonly agreed framework, such as the phenopacket framework, would rapidly deliver significant gains and provide resources to better understand these complex diseases.

To conclude, we have used metaphenomic annotation to structure and standardise 30 years of clinic-pathological data in Parkinsonian syndromes. We have made these resources freely available (https://xip.uclb.com/product/metaphenomic-database-neurodegenerative-disease) in addition to the full codebase to reproduce the entire analysis presented here (https://xip.uclb.com/product/metaphenomic-annotation-of-clinicopathological-parkinsons-disorders). We have used this to begin to build a probabilistic approach to quantify and refine diagnostic precision across Parkinsonian syndromes, providing a foundation for a modular framework that can be flexibly adapted and combined with different tools, techniques, and approaches to more accurately diagnose different Parkinsonian disorders during the early and prodromal phases of the illness.

## Methods

### Literature review

A PubMed search was performed between the dates 1/9/1992 and 1/12/2022 using the keywords “Post-mortem” or “Clinical-pathological” combined with: “Parkinson’s disease”, “Dementia with Lewy Bodies”, “Multiple system atrophy”, “Progressive Supranuclear Palsy”, “Corticobasal degeneration”, “Parkinsonism”. 663 unique articles were identified and reviewed (Q.M., L.N., C.L.). Exclusion criteria included: 1. No post-mortem data; 2. Monogenic disease; 3. No data for main Parkinsonian conditions; 4. Review articles; 5. Not available in English. 6. No basic diagnostic data (i.e., age at diagnosis or disease duration); 7. Unable to annotate (e.g., no extractable data or complex mixed phenotypes). In total, 125 publications (Supplementary Data) were annotated and used for analysis.

### Metaphenomic annotation

Phenopackets (http://phenopackets.org) is a proposed standard for structuring and sharing disease and phenotype data. However, it has primarily been designed for in-person assessment of single cases. Metaphenomic annotation, introduced here, adapts this framework for published phenotyping data, both single subjects and cohorts, following the recommended best practice (see Supplementary Data). It is implemented as a freely available MATLAB toolbox (https://github.com/CPLambert/metaphenomic_annotation_toolbox) for the Statistical Parametric Mapping software (SPM, https://www.fil.ion.ucl.ac.uk/spm/software/spm12/).

### Phenotype and disease ontologies

The “*Human Phenotyping Ontology*” (HPO) and MONDO library of human disease were used throughout. To ensure adequate coverage, we transcribed all clinical phenotype terms from the PD Ontology^9^ to HPO. Any absent terms identified through this work were defined and submitted to the HPO team to provide better coverage.

### Analysis

For all individuals with a diagnosis of sporadic PD, MSA, PSP, DLB and CBD, we extracted: age of onset, age at death, phenotypes, misdiagnosis data, and disease duration. Where possible, onset and duration data were taken relative to symptom onset, but where this was absent, then time from disease diagnosis was used instead (15% of cases). At post-mortem, it is not possible to separate DLB from PD Dementia, and several studies subsume these as “Lewy Body Disease”. Here, we only present results from studies defining PD and DLB as separate cohorts; however, “Lewy Body Disease” results are available via our analysis code. Misdiagnoses falling outside these main disorders were classified as “OTHER”. The misdiagnosis analyses excluded cohort studies that did not report this feature. Co-pathologies, when reported, were captured in the original metaphenomic annotation using the “pathology” and “multidisease” fields (see Supplementary Data for details); however, in this analysis, we focused on the dominant condition present, as defined from the original source publication.

Analysis was done in MATLAB 2021b. Summary statistics were combined using pooled variance and mean^32^. For each dataset, the ratio between sample size versus the total final number in each cohort was used to calculate weighted means. Cohort differences in onset, disease duration and age of death were tested using a Krushkal–Wallis test. If significant, the Wilcoxon rank sum test was used for pair-wise comparisons (Bonferroni *P* < 0.005). To summarise misdiagnosis data, we collapsed each disease into a 2 × 2 confusion matrix where diagnosis in life was framed as the prediction and pathological diagnosis ground truth. In this way, sensitivity, specificity and balanced accuracy were calculated for each diagnosis conditional on the other disorders (detailed in Supplementary Data). To calculate the age of onset and survival probabilities (Figs. 2 and 3), the mean and standard deviation from the corresponding empirical data were used in the MATLAB “*normpdf*” function to fit a normal probability density function spanning the age ranges of 30–100 years for age of onset, and 0–30 years for survival. This returned a range of values for the corresponding Gaussian, which were then sampled for the various examples (e.g., maximum likelihood (Fig.2B) was the age that corresponded to the maximum value of the returned function).

### Probability of disease from phenotypic features

We used a naïve Bayesian classifier approach similar to that proposed for prodromal PD^1^. Additional details on how it was adapted for this work are in the Supplementary Data. In the results, we provide a worked example of how this can be used to refine diagnoses. For this, likelihood ratios (LRs) were calculated from the sensitivity and specificity as follows^33^:

- Positive Likelihood Ratio = Sensitivity/(1-Specificity)
- Negative Likelihood Ratio = (1-Sensitivity)/Specificity

For the example of neurofilament light chain, the published sensitivity was 0.86 and specificity 0.85^12^. These were only available relative to aPD cohorts (MSA/PSP), hence we inverted them (i.e., 1-value) to calculate values for non-aPD groups.

## Supplementary information

Supplementary Information

## Data Availability

To reproduce the analysis and results presented in this work, the original data and code is available via http://xip.uclb.com/product/metaphenomic-annotation-of-clinicopathological-parkinsons-disorders. The metaphenomic annotations created through this work are also part of the metaphenomic database for neurodegenerative disease: http://xip.uclb.com/product/metaphenomic-database-neurodegenerative-disease.
