# Supplementary material for: Refining the diagnostic accuracy of Parkinsonian disorders using metaphenomic annotation of the clinicopathological literature
Source: NPJ Parkinsons Dis. 2025 Nov 10;11:314. doi: 10.1038/s41531-025-01157-y (PMC12603224; doi:10.1038/s41531-025-01157-y)
Supplement: Supplementary file 1 — Supplementary Information [file 41531_2025_1157_MOESM1_ESM.docx]

**Supplementary Material**

Contents

[S1. Supplementary Details: 2](#_Toc200625746)

[S1.1 Adapting Phenopackets for Metaphenomic Annotation 2](#_Toc200625747)

[S1.2 Calculating metrics of diagnostic accuracy 4](#_Toc200625748)

[S1.3 Calculating probability of disease from phenotypic features 4](#_Toc200625749)

[S1.4 Data visualisation 6](#_Toc200625750)

[S2. Supplementary Results: 7](#_Toc200625751)

[S2.1 Summary of data generation 7](#_Toc200625752)

[S2.2 List of annotated clinicopathological studies 7](#_Toc200625753)

[S2.3 Global distribution of cases 11](#_Toc200625754)

[S2.4 Summary of statistical tests 11](#_Toc200625755)

[S2.5 Summary of diagnostic accuracies 12](#_Toc200625756)

[S2.6 Changes in diagnostic accuracy over time 12](#_Toc200625757)

[S3. Worked example of probabilistic modelling: 14](#_Toc200625758)

[S4. Unique Human Phenotyping Ontology terms 15](#_Toc200625759)

[S5. Supplementary References: 20](#_Toc200625760)

# **S1. Supplementary Details:**

## **S1.1 Adapting Phenopackets for Metaphenomic Annotation**

The basic Phenopackets ^1^ structure uses a protobuf schema which is "*a language-neutral, platform-neutral extensible mechanism for serializing structured data*”. This is based around so-called “*building blocks*” which are standardised fields that can be used to structure information. We adapted it to aggregate phenotype meta-analytic data. Specifically, the following changes were added to this framework: we added “*Publication*” as a new top-level field and took the PMID structured fields as building blocks for this entry. This process allowed us to fully automate field annotation by providing the .csv download data from Pubmed searches. In addition to this, we added several new “*building-blocks*” to the existing top-level field cohort. These changes/adaptations are summarised in table 1. Because published cohort studies may include several cohorts, we added a suffix the main top-level cohort field _[number], but where single case reports were extractable defaulted native to the phenopacket standard. We identified the distinction by labelling the outputs either “*phenopacket*” or “*metaphenome-annot”.* Regarding the misdiagnosis field, we identified two different *types* of misdiagnoses that may be described in a cohort study, depending on whether the analysis is looking forwards or backwards in time. The former involves a published cohort being labelled by their diagnosis in life but the post-mortem shows otherwise, which we label as a *prospective misdiagnosis*. In the latter case, a cohort may be identified at post-mortem with the same "gold standard" diagnosis, but review of the historical records reveals a different diagnosis in life, which we label a *retrospective misdiagnosis*. The distinction allows us to subsequently combine both types of data. We also added a pathology block, to capture reported histopathological data. This embedded Braak AD stage, ^2^ Thala Beta stage, ^3^ Plaque Score (qualitative), MSA pathological subtype, Lewy Body Disorder subtype and Likelihood of DLB into the main toolbox but could also manually define other schemes. Finally, we added a dual diagnosis field where mixed proteinopathies were clearly identified and labelled (however in the future this may be subsumed by the pathology field). All cohort data only included the total number with the post-mortem confirmed diagnoses (i.e., misdiagnosis data was excluded/subtracted from these figures at the time of annotation and entered separately in the misdiagnosis fields).

Because our objective was to produce machine readable files that also align with the Brain Imaging Dataset Standard ^4^ (BIDS, https://bids-specification.readthedocs.io/en/stable/), we chose .json as the default output. However, it is possible to convert these to protobuf where required. The resulting output filename was structured as follows:

- Metaphenome file: [PMID]_[FIRST-AUTHOR]_[PUB-DATE]_metaphenome-annot.json

- Phenopacket file: [PMID]_[FIRST-AUTHOR]_[PUB-DATE]_phenopacket-sub-[no].json

Where PMID is the pubmed ID, and publication date provided in the form yyyymmdd. Finally, for all summary statistics, if the median and confidence intervals were provided, they were also converted to mean and standard deviation using the method described by Wan et al 2014 to facilitate second-level data aggregation. ^5^

To note, since the analysis for this work was concluded there was a major update to the phenopacket schema which added their own “*measurement*” fields (absent in version 1.0 when changes in table 1 were specified). Whilst we have attempted to align as closely as possible with the original phenopacket schema, the metaphenomic implementation has been designed with a slightly different purpose in mind (i.e., optimised for cohort data aggregation rather than single case descriptions) and retrofitting the updated measurement field would require a substantial update to the toolbox and underlying code. Therefore, for the purposes of this work we have retained our original framework, and plan to harmonise these differences in future updates.

**Supplementary Table 1 -** **Summary of changes to the phenopacket schema**

## **S1.2 Calculating metrics of diagnostic accuracy**

In this work, we were interested in pooling information across different Parkinsonian disorders and temporally directed analyses (post-mortem compared to life-time diagnosis and visa versa) to try and provide more granular insights into overall diagnostic accuracy. To summarise these, we collapsed the data for each disease into a 2×2 confusion matrix, where the diagnosis in life was framed as the prediction, and pathological diagnosis as the ground truth. In this way, we could calculate the corresponding sensitivity, specificity and balanced accuracy for each clinical diagnosis, conditional on all of the main Parkinsonian disorders.


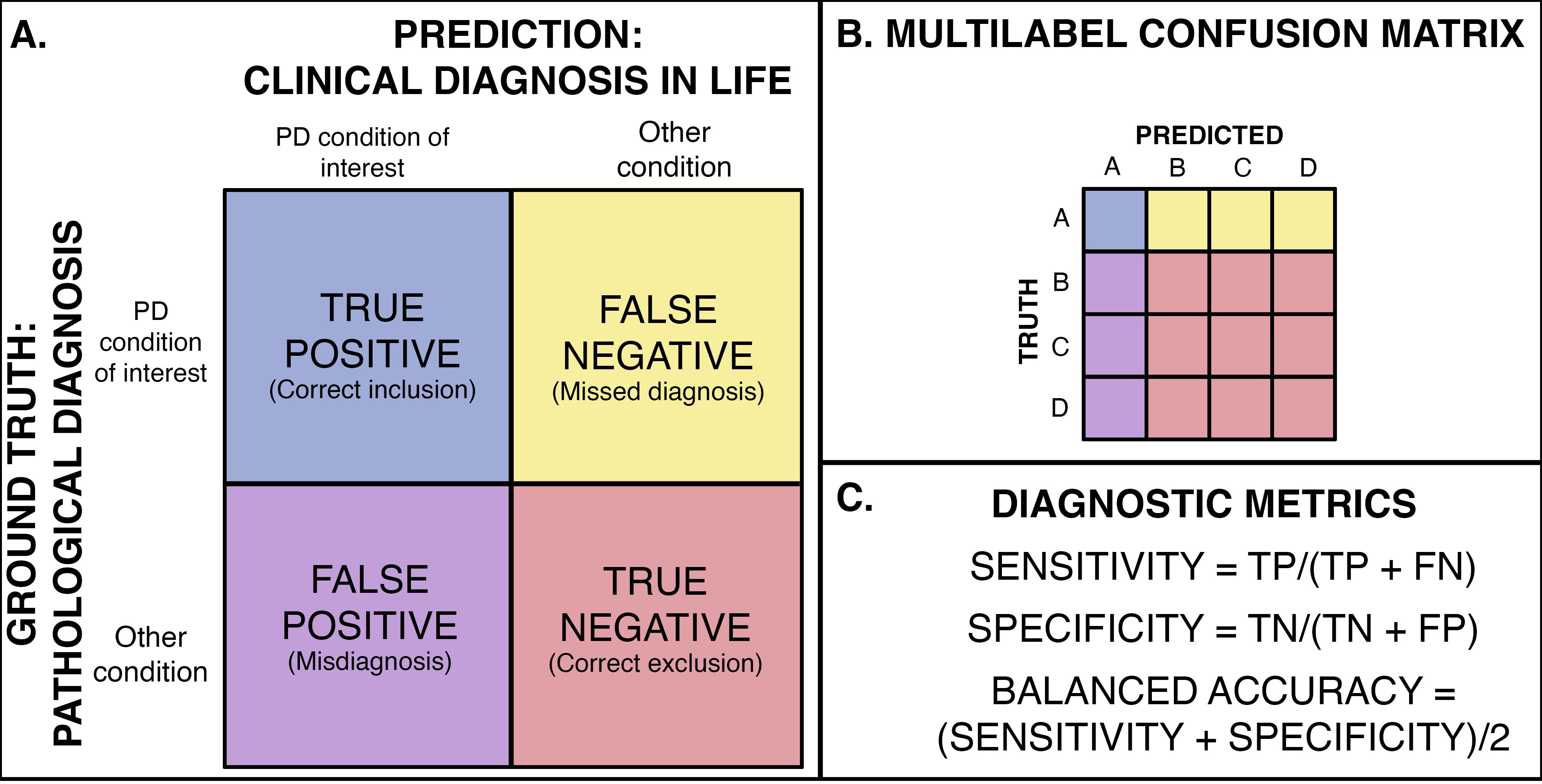


**Supplementary Figure 1 – Diagnostic accuracy calculations:** ***A.*** *Diagnosis definitions mapped to 2×2 confusion matrix;* ***B.*** *Collapsing multiple categories into 2×2 confusion matrix to calculate summary metrics, in this example the disease of interest is “A”;* ***C.*** *Calculation of diagnostic accuracy metrics.*

## **S1.3 Calculating probability of disease from phenotypic features**

We adopted a naïve Bayesian classifier approach similar to the probability of prodromal Parkinson’s disease approach. ^6^ The advantage is that it allows diagnostic information to be sequentially added and used to update pretest probability of disease (P) given new information. Furthermore, providing our results in this format also means they can easily be used by other similar classifiers allowing *in vivo* models to incorporate diagnostic uncertainty and leverage post-mortem defined likelihood ratios (LR). Finally, by providing the original underlying data in a structured, reusable, machine-readable format, these probabilities can be rapidly and iteratively updated as new results emerge. This approach has been described elsewhere ^6^ but has been summarized below for clarity:

$${LR}_{total}=\prod_{1}^{n} LR$$

$${ODDS}_{post}=P*{LR}_{total}$$

$$P_{disease}=\frac{{ODDS}_{post}}{(1+{ODDS}_{post})}$$

Where:

- P = Pre-test probability
- LR = Likelihood ratios for features of interest
- n = Total number of observed features with LRs
- LR_total_ = Pooled likelihood ratio
- ODDS_post_ = Post-test odds
- P_disease_ = Probability of disease

Because the objective was to provide a means to quantify diagnostic precision in an individual presenting with Parkinsonism, we did not incorporate the population prevalence into our model, but simply calculated our pre-test odds (P) based on age at presentation directly from the post-mortem data.

There was marked variability in the literature as to when an illness’ various features (phenotypic traits) were described, what was described or omitted, how they were categorized, and the ontologies used. For simplicity, in this analysis a phenotype was included if it occurred at any point during the illness, and the sample population was calculated for each phenotype by only counting studies where that feature was described. Overall, 88% of studies provided some data that could be extracted, providing 4076 features. After review, these could be collapsed to 246 unique HPO terms. This exercise resulted in a number of new terms and suggested modifications to the existing HPO framework improve coverage for these movement disorders. Supplementary figure 2 provides an example of this framework for the human phenotyping ontology (HPO) term Parkinsonism:


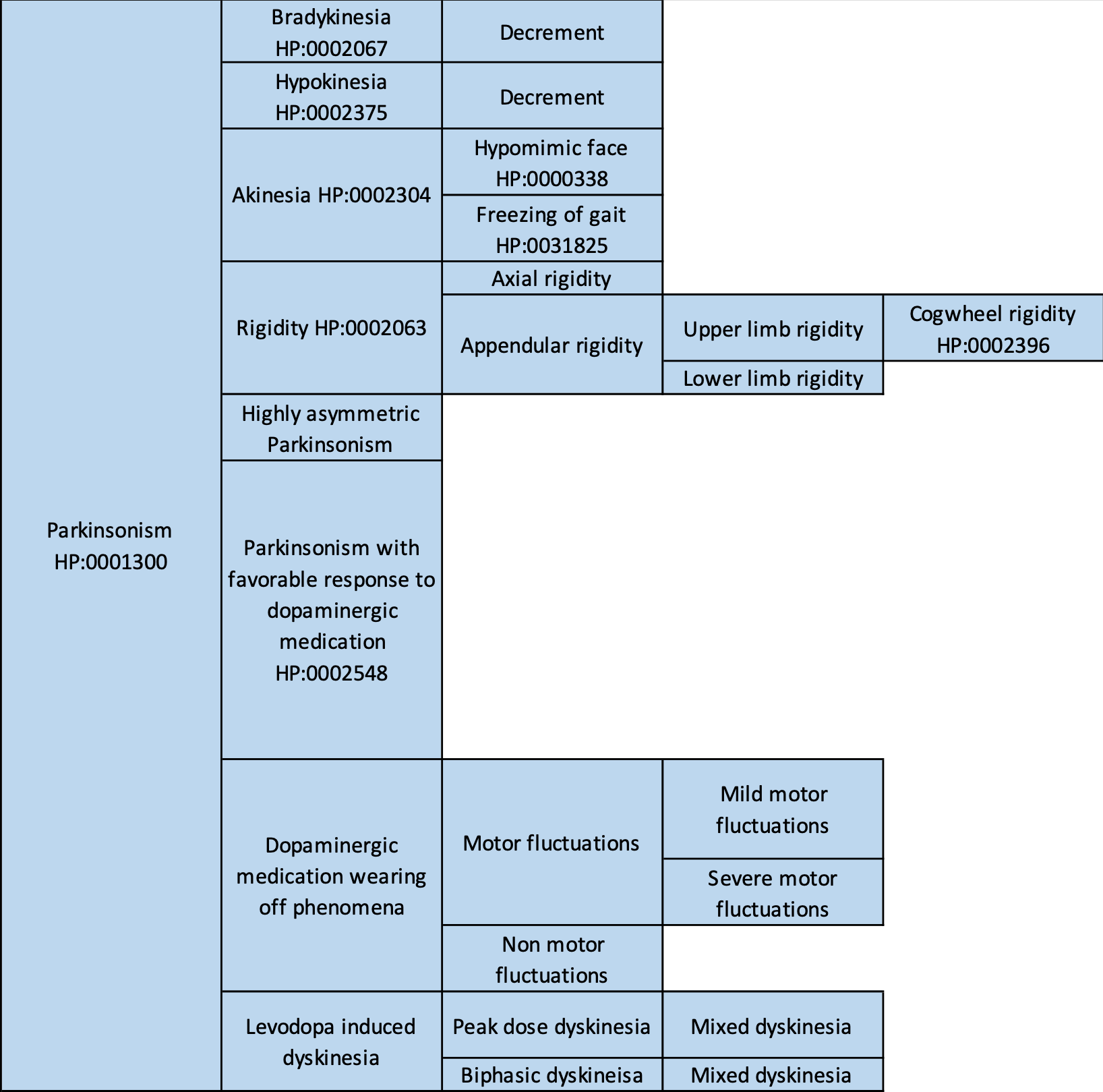


**Supplementary Figure 2 - HPO Inheritance principle optimised for Parkinson’s disease** - P*art of Abnormality of movement HP:0100022 shown. Note all terms with missing HPO IDs have been defined and submitted to HPO to provide better coverage Parkinsonian for movement* *disorders.*

Each unique HPO term was then mapped to the broader HPO hierarchical framework, which organises terms as a directed acyclic graph where each sub-term (child) represents a more specific or limited instance of its parent term(s) and can be connected by a *“is-a”* (>) relationship, for example:

*Bradykinesia* > *Parkinsonism* > *Diminished Movements* > *Abnormality of Movement*

This means that incomplete coverage of low-frequency phenotypes still contribute to provide better coverage further up the HPO hierarchy. Because this approach combines both single case studies and group cohort data, propagating information up the HPO tree represents a challenge because, in contrast to single subject observations, a negative observation in one of the child terms does not necessarily mean the parent term was absent – Taking only the positive observations risked up-weighting rare phenotypes, whereas summing both the present and absent phenotypes present in all child terms risked down-weighting more common higher-level parent phenotypes. Because the objective was to create likelihood ratios of one disease relative to another, we were able to test both approaches by calculating the LR for every HPO term in the hierarchy, across all the diseases, and reviewing the top-ranked terms (Figure 7). We found summing both present and absent observations provided the best solution, provided observations were present for eight or more individuals for the main group, and applied this to the entire HPO tree to calculate positive and negative likelihood ratios for every phenotypic feature between the five main conditions (PD, MSA, PSP, DLB, CBD). We removed any LRs where the denominator was zero in this work (i.e., LR = infinity) – Whilst these may reflect pathognomonic clinical signs, given the stark variability in what clinical phenotypes were reported in the clinic-pathological literature, we could confidently rank these as such in this work, as some of the results were clearly artefactual due to under-reporting and incomplete coverage. This limitation could be offset in the future by incorporating more detailed observations from other cohorts (e.g., *in vivo* observational studies).

## **S1.4 Data visualisation**

All data visualisation code was created by CL and implemented in MATLAB - Gaussian probability density, cumulative distribution functions and heatplots were generated using inbuilt MATLAB functions. The RainCloud plots were adapted from Allen. ^7^. The riverplots used to summarise change in diagnosis were adapted from the “Sankey Diagram” code available via MATLAB central. ^8^ The colorbrewer palattes were used. ^9^ All data-visualisation code has been made available with the main analysis code at: [https://xip.uclb.com/product/metaphenomic-annotation-of-clinicopathological-parkinsons-disorders](https://eur01.safelinks.protection.outlook.com/?url=https%3A%2F%2Fxip.uclb.com%2Fproduct%2Fmetaphenomic-annotation-of-clinicopathological-parkinsons-disorders&data=05%7C02%7Cchristian.lambert%40ucl.ac.uk%7C53fbca72c69a40f919c608dd9de1bf0f%7C1faf88fea9984c5b93c9210a11d9a5c2%7C0%7C0%7C638840315086442294%7CUnknown%7CTWFpbGZsb3d8eyJFbXB0eU1hcGkiOnRydWUsIlYiOiIwLjAuMDAwMCIsIlAiOiJXaW4zMiIsIkFOIjoiTWFpbCIsIldUIjoyfQ%3D%3D%7C0%7C%7C%7C&sdata=dNHZOm%2F1ZfAnFX0994s%2B3%2FsvTivj%2FyUwQ%2BXZz8Kf5iA%3D&reserved=0)

# **S2. Supplementary Results:**

**
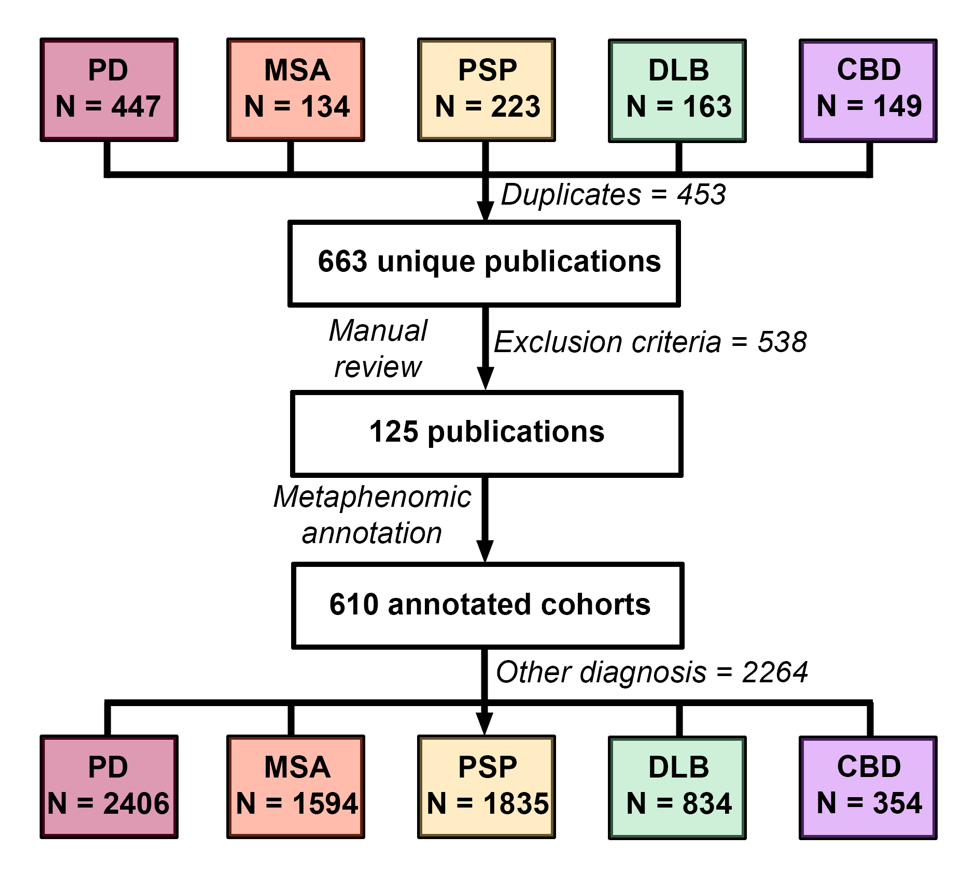
****S2.1 Summary of data generation:**

**Supplementary Figure 3: Summary of data generation** - Pubmed search between the dates 1/9/1992 – 1/12/2022 using the keywords *“Post-mortem”* or *“Clinical-pathological”* combined with each condition shown. Top row shows number of publications and filtering process. Bottom row show the total number of cases in each cohort generated through the metaphenomic annotation. Note, of these 9287 cases only 5748 provided misdiagnosis data as detailed in the main text.

**S2.2 List of annotated clinicopathological studies**

1, Sobue G,1992: Somatic motor efferents in multiple system atrophy with autonomic failure: a clinico-pathological study

2, Hughes AJ,1992: Accuracy of clinical diagnosis of idiopathic Parkinson's disease: a clinico-pathological study of 100 cases

3, Jankovic J,1993: What is it? Case 1, 1993: parkinsonism, dysautonomia, and ophthalmoparesis

4, Churchyard A,1993: Dopa resistance in multiple-system atrophy: loss of postsynaptic D2 receptors

5, ,1993: Case records of the Massachusetts General Hospital. Weekly clinicopathological exercises. Case 46-1993. A 75-year-old man with right-sided rigidity, dysarthria, and abnormal gait

6, Wenning GK,1994: Clinical features and natural history of multiple system atrophy. An analysis of 100 cases

7, Harris CP,1994: Depression followed by dementia and disordered movement. Clinicopathologic correlation

8, de Vos RA,1995: 'Lewy body disease': clinico-pathological correlations in 18 consecutive cases of Parkinson's disease with and without dementia

9, Wenning GK,1995: Clinicopathological study of 35 cases of multiple system atrophy

10, Collins SJ,1995: Progressive supranuclear palsy: neuropathologically based diagnostic clinical criteria

11, Litvan I,1996: Validity and reliability of the preliminary NINDS neuropathologic criteria for progressive supranuclear palsy and related disorders

12, Litvan I,1996: Natural history of progressive supranuclear palsy (Steele-Richardson-Olszewski syndrome) and clinical predictors of survival: a clinicopathological study

13, Verny M,1996: Progressive supranuclear palsy: a clinicopathological study of 21 cases

14, Bergeron C,1996: Unusual clinical presentations of cortical-basal ganglionic degeneration

15, Litvan I,1997: Which clinical features differentiate progressive supranuclear palsy (Steele-Richardson-Olszewski syndrome) from related disorders? A clinicopathological study

16, Schneider JA,1997: Corticobasal degeneration: neuropathologic and clinical heterogeneity

17, ,1997: Case records of the Massachusetts General Hospital. Weekly clinicopathological exercises. Case 26-1997. A 64-year-old man with progressive dementia, seizures, and unstable gait

18, Tsuchiya K,1997: Distribution of cerebral cortical lesions in corticobasal degeneration: a clinicopathological study of five autopsy cases in Japan

19, Wenning GK,1998: Natural history and survival of 14 patients with corticobasal degeneration confirmed at postmortem examination

20, Boeve BF,1999: Pathologic heterogeneity in clinically diagnosed corticobasal degeneration

21, Wenning GK,1999: Time course of symptomatic orthostatic hypotension and urinary incontinence in patients with postmortem confirmed parkinsonian syndromes: a clinicopathological study

22, Wenning GK,1999: Progression of falls in postmortem-confirmed parkinsonian disorders

23, Grimes DA,1999: Dementia as the most common presentation of cortical-basal ganglionic degeneration

24, Litvan I,1999: Clinicopathologic case report. Dementia with Lewy bodies (DLB)

25, Hohl U,2000: Diagnostic accuracy of dementia with Lewy bodies

26, Armstrong RA,2000: A quantitative study of the pathological lesions in the neocortex and hippocampus of twelve patients with corticobasal degeneration

27, Tsuchiya K,2000: Constant involvement of the Betz cells and pyramidal tract in multiple system atrophy: a clinicopathological study of seven autopsy cases

28, Mimura M,2001: Corticobasal degeneration presenting with nonfluent primary progressive aphasia: a clinicopathological study

29, Müller J,2001: Progression of dysarthria and dysphagia in postmortem-confirmed parkinsonian disorders

30, Mann DM,2001: Anosmia in dementia is associated with Lewy bodies rather than Alzheimer's pathology

31, Vitaliani R,2002: The pathology of the spinal cord in progressive supranuclear palsy

32, Hughes AJ,2002: The accuracy of diagnosis of parkinsonian syndromes in a specialist movement disorder service

33, Müller J,2002: Freezing of gait in postmortem-confirmed atypical parkinsonism

34, Josephs KA,2002: A clinicopathological study of vascular progressive supranuclear palsy: a multi-infarct disorder presenting as progressive supranuclear palsy

35, Harding AJ,2002: Clinical correlates of selective pathology in the amygdala of patients with Parkinson's disease

36, Poewe W,2002: The differential diagnosis of Parkinson's disease

37, Birdi S,2002: Progressive supranuclear palsy diagnosis and confounding features: report on 16 autopsied cases

38, Mochizuki A,2003: Progressive supranuclear palsy presenting with primary progressive aphasia--clinicopathological report of an autopsy case

39, Colosimo C,2003: Lewy body cortical involvement may not always predict dementia in Parkinson's disease

40, Osaki Y,2004: Accuracy of clinical diagnosis of progressive supranuclear palsy

41, Lezcano E,2004: Parkinson's disease-like presentation of multiple system atrophy with poor response to STN stimulation: a clinicopathological case report

42, Schlossmacher MG,2004: Case records of the Massachusetts General Hospital. Weekly clinicopathological exercises. Case 27-2004. A 79-year-old woman with disturbances in gait, cognition, and autonomic function

43, Ozawa T,2004: The spectrum of pathological involvement of the striatonigral and olivopontocerebellar systems in multiple system atrophy: clinicopathological correlations

44, Tsuchiya K,2005: Constant and severe involvement of Betz cells in corticobasal degeneration is not consistent with pyramidal signs: a clinicopathological study of ten autopsy cases

45, Josephs KA,2005: Extending the clinicopathological spectrum of neurofilament inclusion disease

46, Williams DR,2005: Characteristics of two distinct clinical phenotypes in pathologically proven progressive supranuclear palsy: Richardson's syndrome and PSP-parkinsonism

47, Tsuboi Y,2005: Increased tau burden in the cortices of progressive supranuclear palsy presenting with corticobasal syndrome

48, Halliday GM,2005: A comparison of degeneration in motor thalamus and cortex between progressive supranuclear palsy and Parkinson's disease

49, Papapetropoulos S,2005: Natural history of progressive supranuclear palsy: a clinicopathologic study from a population of brain donors

50, Josephs KA,2005: Atypical progressive supranuclear palsy underlying progressive apraxia of speech and nonfluent aphasia

51, Josephs KA,2006: Clinicopathological and imaging correlates of progressive aphasia and apraxia of speech

52, Kłodowska-Duda G,2006: Corticobasal degeneration -- clinico-pathological considerations

53, Murray R,2007: Cognitive and motor assessment in autopsy-proven corticobasal degeneration

54, Kempster PA,2007: Patterns of levodopa response in Parkinson's disease: a clinico-pathological study

55, Compta Y,2007: Long lasting pure freezing of gait preceding progressive supranuclear palsy: a clinicopathological study

56, Facheris MF,2008: Pure akinesia as initial presentation of PSP: a clinicopathological study

57, Jellinger KA,2008: Different tau pathology pattern in two clinical phenotypes of progressive supranuclear palsy

58, Lladó A,2008: Clinicopathological and genetic correlates of frontotemporal lobar degeneration and corticobasal degeneration

59, O'Sullivan SS,2008: Clinical outcomes of progressive supranuclear palsy and multiple system atrophy

60, Kalaitzakis ME,2009: Dementia and visual hallucinations associated with limbic pathology in Parkinson's disease

61, Brooks D,2009: Intralaminar nuclei of the thalamus in Lewy body diseases

62, Kanazawa M,2009: Cerebellar involvement in progressive supranuclear palsy: A clinicopathological study

63, Rajput AH,2009: Course in Parkinson disease subtypes: A 39-year clinicopathologic study

64, Selikhova M,2009: A clinico-pathological study of subtypes in Parkinson's disease

65, Sabbagh MN,2009: Parkinson disease with dementia: comparing patients with and without Alzheimer pathology

66, Molano J,2010: Mild cognitive impairment associated with limbic and neocortical Lewy body disease: a clinicopathological study

67, Kempster PA,2010: Relationships between age and late progression of Parkinson's disease: a clinico-pathological study

68, Ozawa T,2010: The phenotype spectrum of Japanese multiple system atrophy

69, Ling H,2010: Does corticobasal degeneration exist? A clinicopathological re-evaluation

70, Espay AJ,2011: Rapidly progressive atypical parkinsonism associated with frontotemporal lobar degeneration and motor neuron disease

71, Snowden JS,2011: The clinical diagnosis of early-onset dementias: diagnostic accuracy and clinicopathological relationships

72, Kouri N,2011: Neuropathological features of corticobasal degeneration presenting as corticobasal syndrome or Richardson syndrome

73, Iodice V,2012: Autopsy confirmed multiple system atrophy cases: Mayo experience and role of autonomic function tests

74, Magdalinou NK,2013: Normal pressure hydrocephalus or progressive supranuclear palsy? A clinicopathological case series

75, Iwasaki Y,2013: An autopsied case of progressive supranuclear palsy presenting with cerebellar ataxia and severe cerebellar involvement

76, Boeve BF,2013: Clinicopathologic correlations in 172 cases of rapid eye movement sleep behavior disorder with or without a coexisting neurologic disorder

77, Shim YS,2013: Clinicopathologic study of Alzheimer's disease: Alzheimer mimics

78, Menšíková K,2013: Progressive supranuclear palsy phenotype mimicking synucleinopathies

79, Fujioka S,2013: Similarities between familial and sporadic autopsy-proven progressive supranuclear palsy

80, Joutsa J,2014: Diagnostic accuracy of parkinsonism syndromes by general neurologists

81, Figueroa JJ,2014: Multiple system atrophy: prognostic indicators of survival

82, Adler CH,2014: Low clinical diagnostic accuracy of early vs advanced Parkinson disease: clinicopathologic study

83, Jacobson SA,2014: Plaques and tangles as well as Lewy-type alpha synucleinopathy are associated with formed visual hallucinations

84, Ikeda C,2014: Corticobasal degeneration initially developing motor versus non-motor symptoms: a comparative clinicopathological study

85, Respondek G,2014: The phenotypic spectrum of progressive supranuclear palsy: a retrospective multicenter study of 100 definite cases

86, Zhu MW,2015: Typical or atypical progressive supranuclear palsy: a comparative clinicopathologic study of three Chinese cases

87, Cykowski MD,2015: Expanding the spectrum of neuronal pathology in multiple system atrophy

88, Koga S,2015: When DLB, PD, and PSP masquerade as MSA: an autopsy study of 134 patients

89, Virmani T,2015: Clinicopathological characteristics of freezing of gait in autopsy-confirmed Parkinson's disease

90, Iacono D,2015: Parkinson disease and incidental Lewy body disease: Just a question of time?

91, Xie T,2015: Comparison of clinical features in pathologically confirmed PSP and MSA patients followed at a tertiary center

92, Beach TG,2016: Prevalence of Submandibular Gland Synucleinopathy in Parkinson's Disease, Dementia with Lewy Bodies and other Lewy Body Disorders

93, Koga S,2016: Cerebellar ataxia in progressive supranuclear palsy: An autopsy study of PSP-C

94, Kurz C,2016: An autopsy-confirmed case of progressive supranuclear palsy with predominant postural instability

95, Adamowicz DH,2017: Hippocampal α-Synuclein in Dementia with Lewy Bodies Contributes to Memory Impairment and Is Consistent with Spread of Pathology

96, Rajput AH,2017: Octogenarian parkinsonism - Clinicopathological observations

97, Walker L,2017: Quantitative neuropathology: an update on automated methodologies and implications for large scale cohorts

98, Suemoto CK,2017: Neuropathological diagnoses and clinical correlates in older adults in Brazil: A cross-sectional study

99, Turcano P,2017: Clinicopathologic discrepancies in a population-based incidence study of parkinsonism in olmsted county: 1991-2010

100, Jung Y,2018: Clinicopathological and (123)I-FP-CIT SPECT correlations in patients with dementia

101, Roudil J,2018: Influence of Lewy Pathology on Alzheimer's Disease Phenotype: A Retrospective Clinico-Pathological Study

102, De Pablo-Fernández E,2019: Prognosis and Neuropathologic Correlation of Clinical Subtypes of Parkinson Disease

103, Stejskalova Z,2019: Pyramidal system involvement in progressive supranuclear palsy - a clinicopathological correlation

104, Miki Y,2019: Improving diagnostic accuracy of multiple system atrophy: a clinicopathological study

105, Jabbari E,2019: The genetic and clinico-pathological profile of early-onset progressive supranuclear palsy

106, Vergouw LJM,2020: Dementia With Lewy Bodies: A Clinicopathologic Series of False-positive Cases

107, Jabbari E,2020: Diagnosis Across the Spectrum of Progressive Supranuclear Palsy and Corticobasal Syndrome

108, Knox MG,2020: Neuropathological Findings in Parkinson's Disease With Mild Cognitive Impairment

109, Koga S,2020: Clinicopathologic and genetic features of multiple system atrophy with Lewy body disease

110, Smirnov DS,2020: Cognitive decline profiles differ in Parkinson disease dementia and dementia with Lewy bodies

111, Koga S,2020: Cerebrovascular pathology and misdiagnosis of multiple system atrophy: An autopsy study

112, Homma T,2020: Cerebral white matter tau-positive granular glial pathology as a characteristic pathological feature in long survivors of multiple system atrophy

113, Boes S,2020: Dementia with Lewy bodies presenting as Logopenic variant primary progressive Aphasia

114, Longardner K,2020: Orthostatic hypotension preceding dementia with Lewy bodies by over 15 years: a clinicopathologic case report

115, Hansen D,2021: Novel clinicopathological characteristics differentiate dementia with Lewy bodies from Parkinson's disease dementia

116, Ishida C,2021: Effectiveness of Levodopa in Patients with Multiple System Atrophy and Associated Clinicopathological Features

117, Zhang S,2021: Case Report of a pathologically confirmed vascular parkinsonism with early cognitive impairment and Behavioral disturbance

118, Homma T,2021: Digital mapping of Lewy bodies and neurites in alpha-synuclein stained large cerebral hemispheric sections from three patients with dementia with Lewy bodies showing psychotic manifestations: A pilot study

119, Crosiers D,2021: Cerebellar ataxia in progressive supranuclear palsy: a clinico-pathological case report

120, Chatterjee A,2021: Clinico-pathological comparison of patients with autopsy-confirmed Alzheimer's disease, dementia with Lewy bodies, and mixed pathology

121, Donlon E,2021: Braak's Unfinished Hypothesis: A Clinicopathological Case Report of α-Synuclein Peripheral Neuropathy Preceding Parkinsonism by 20 Years

122, Kawakatsu S,2021: Clinicopathological heterogeneity of Alzheimer's disease with pure Alzheimer's disease pathology: Cases associated with dementia with Lewy bodies, very early-onset dementia, and primary progressive aphasia

123, Natera-Villalba E,2021: Eye-of-the-Tiger Sign with an Unexpected Pathological Diagnosis

124, Lin CR,2022: Clinicopathological correlates of pyramidal signs in multiple system atrophy

125, Horimoto Y,2022: A descriptive study of Parkinson disease and atypical parkinsonisms in the Annuals of the Pathological Autopsy Cases in Japan

## **S2.3 Global distribution of cases**


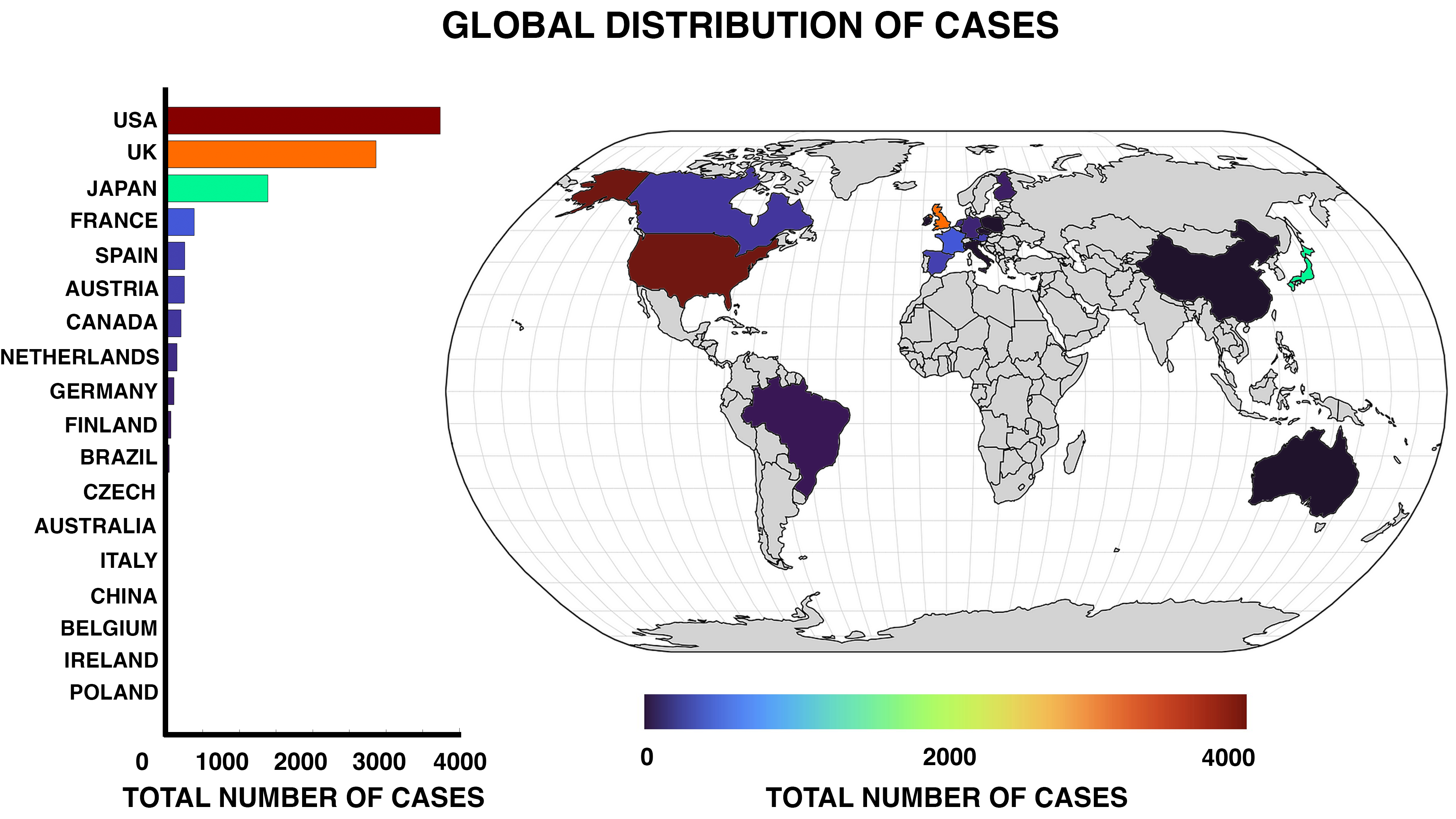


**Supplementary Figure 4: Global distribution of cases** – Number of clinicopathological cases contributed by each country.

## **S2.4 Summary of statistical tests**


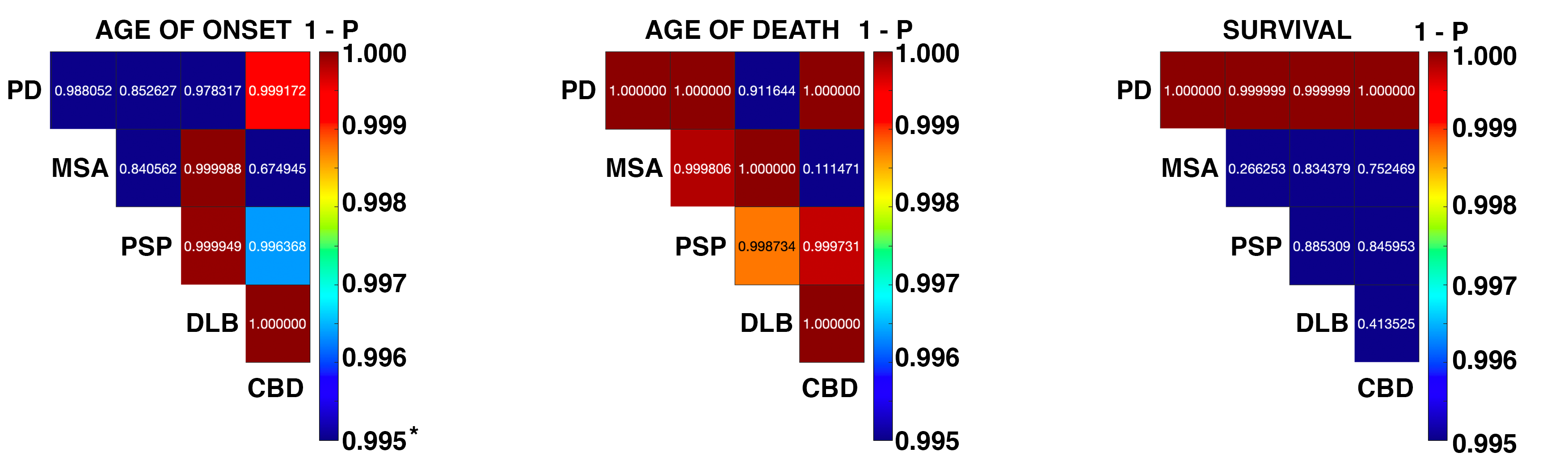


**Supplementary Figure 5: Summary of statistical test results** – Heatmaps of one minus p value summarising each of the pairwise tests for age of onset, age of death and survival (disease duration), thresholded at P < 0·005 (bonferroni corrected value).

## **S2.5 Summary of diagnostic accuracies**

Accuracy metrics pooling mis- and missed diagnoses data across all conditions:

**Supplementary Table 2 -** **Summary of diagnostic accuracies over all conditions**

Accuracy metrics pooling mis- and missed diagnoses data combining PD and DLB into a single lewy-body disorder category:

**Supplementary Table 3 -** **Summary of diagnostic accuracy for Lewy Body Disorders**

## **S2.6 Changes in diagnostic accuracy over time**

We extracted the absolute counts (true positive, true negative, false positive, false negative) in five-yearly time bins from 1992 through to 2022, and recalculated the diagnostic metrics (Supplementary Figure 1) for each time window. This is summarized in supplementary figure 6. This shows that there is no clear correlation with time (Spearman’s correlation), particularly for PD, MSA and PSP. The early papers with DLB are biased by very low numbers of published cases resulting in implausibly high diagnostic accuracies, but since 2010 the number of published cases have increased and the balanced accuracy has dropped accordingly. CBD shows a significant drop following 2012 – This may correspond to the publication of “*Criteria for the diagnosis of corticobasal degeneration*” (Armstrong et al., 2013), that established the broader phenotypic spectrum (in life) of clinicopathologically defined CBD and resulted in greater recognition of the true misdiagnosis rates. Alternatively, there were less reported cases overall, and it may simply be a bias following this publication. However, pooling the absolute counts over time will help offset variation over time and different centres/investigators, and the overall diagnostic accuracy would be expected to converge upon the true mean given sufficiently large samples sizes.


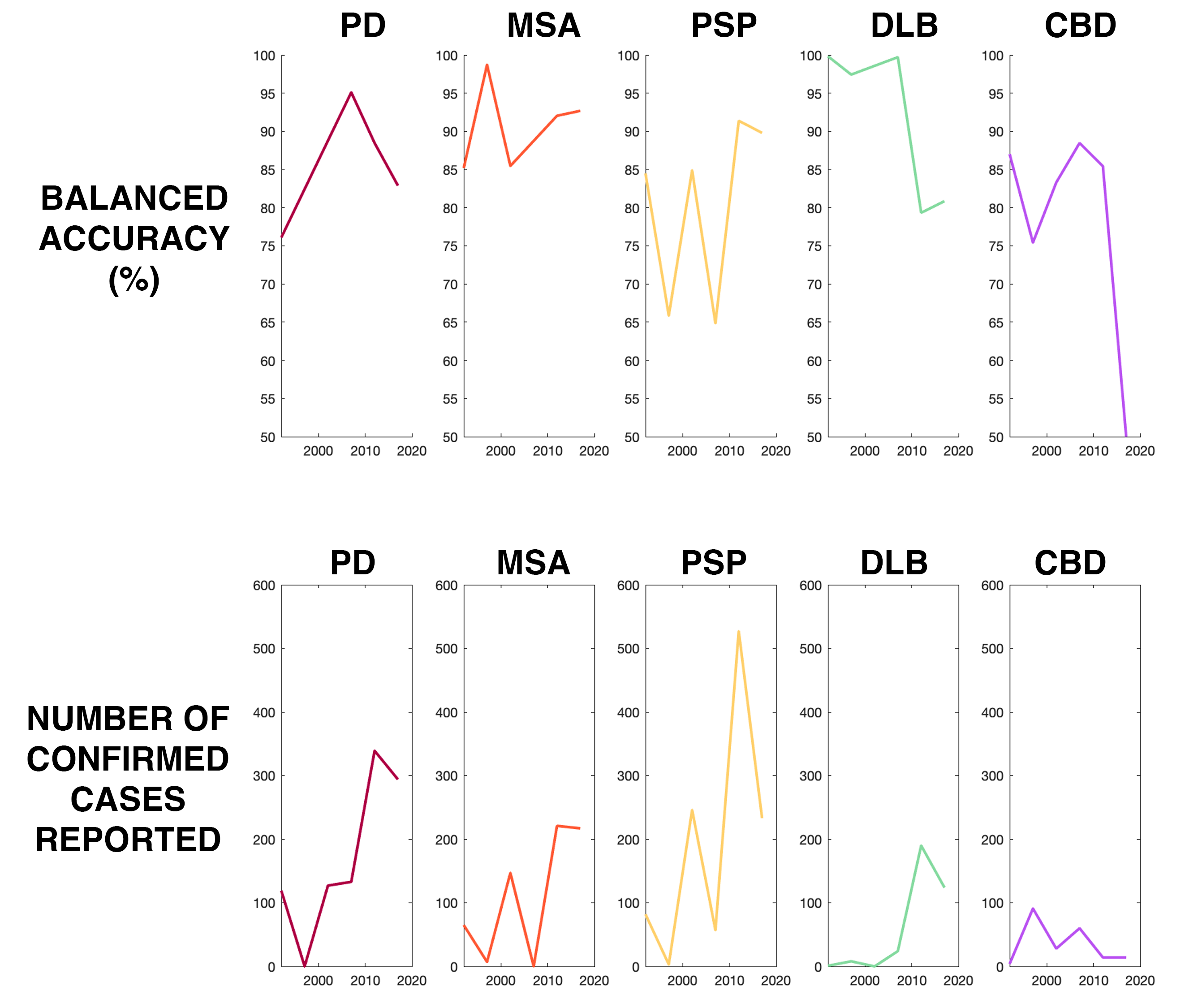


**Supplementary Figure 6: Changes in diagnostic accuracy over time**

# **S3. Worked example of probabilistic modelling:**

**
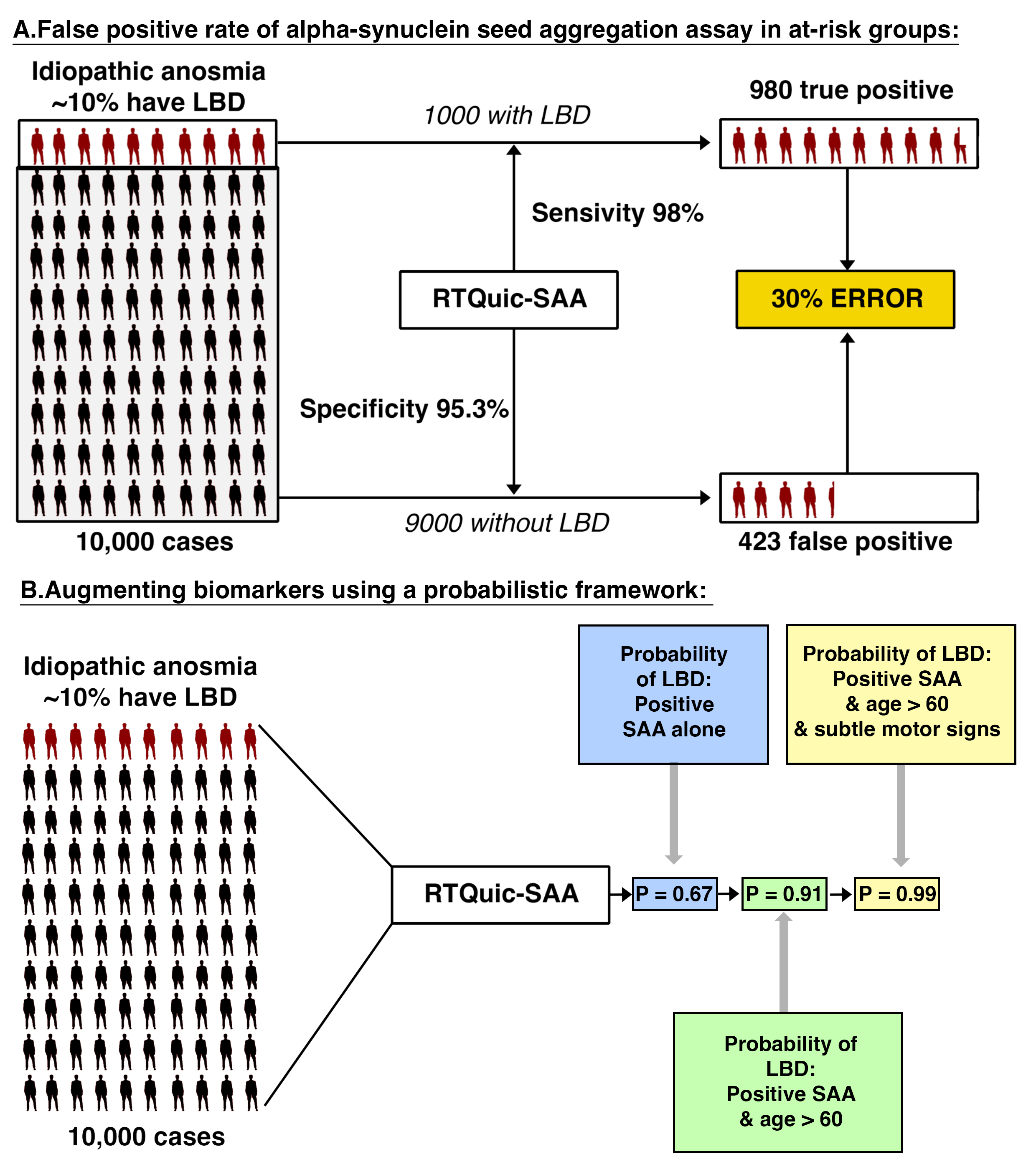
**

**Supplementary Figure 7 - Worked example of probabilistic modelling:** A. Population level application of the alpha-synuclein seed aggregation assay to an at-risk prodromal group: The false positive rate is conflated due to the difference in disease prevalence at a population level, which significantly scales the false positive results (i.e. many more people don’t have the disease). This shows that it is unlikely that any prodromal diagnostic pathway will rely on one single test or modality. B. Augmenting biomarkers using a probabilistic framework: Using the same biomarker example above, it has now been framed in terms of probability of disease, and then shows how a significant increase in likelihood can be achieved through relatively simple and easy to acquire clinical observations. Similarly, any other conceivable biomarker, measure or observation can be easily integrated and used in this framework in an additive fashion.


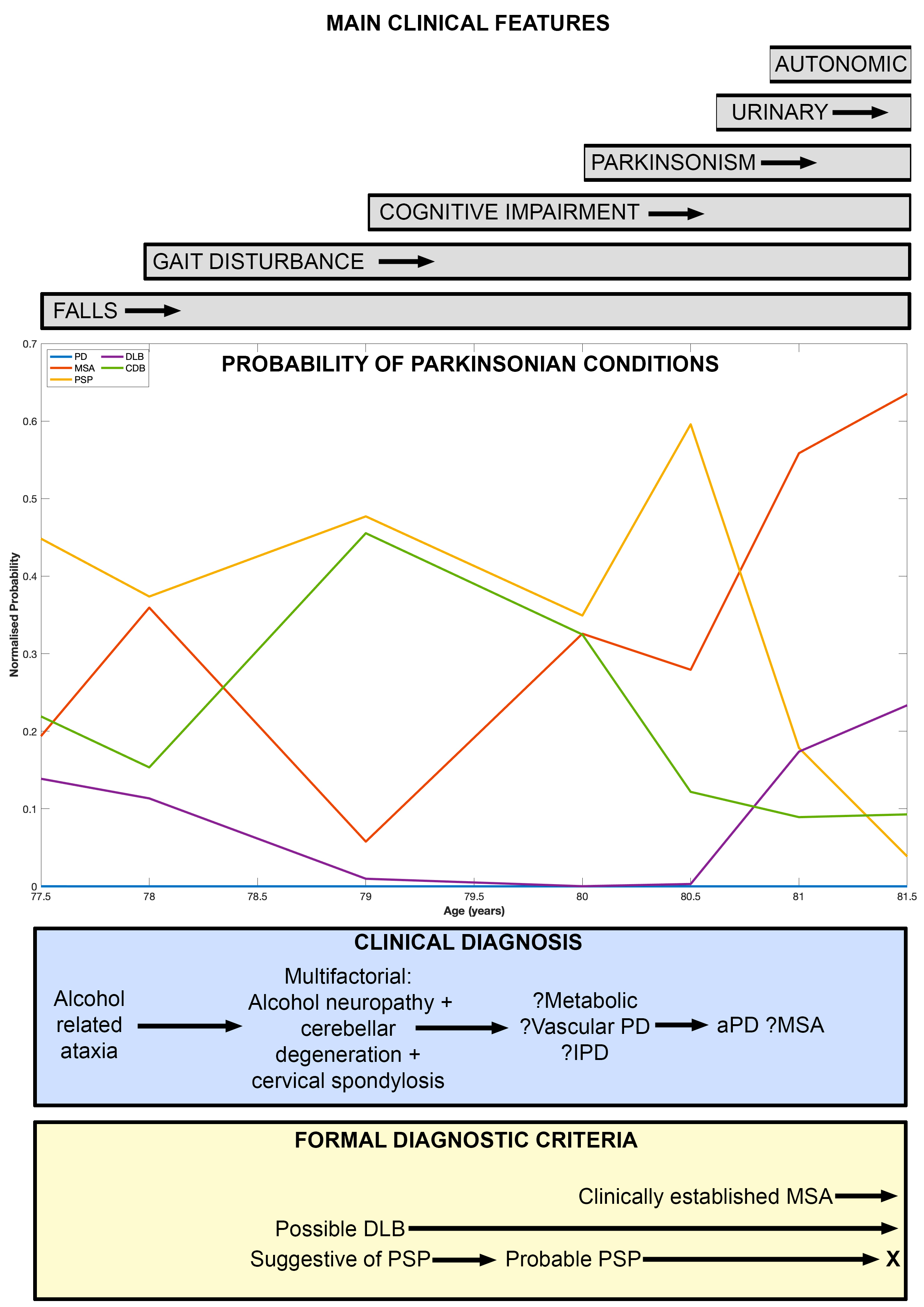


**Supplementary Figure 8 - Worked example of longitudinal probabilistic modelling:** Likelihood ratios over all possible phenotypes were re-calculated holding out a published clinicopathological MSA case report from NEJM (Case 27-2004)^10^. Probabilities for every clinical interaction, from prodrome to death, were calculated based on all available reported features and normalised to sum to one over all conditions. Boxes above also show a summary of the main clinical features (grey), clinical impression (blue) and latest formal diagnostic criteria (yellow). This shows the probabilistic approach arrived at the correct answer but also reveals that the diagnostic confusion arose with the onset of cognitive symptoms at age 79. In contrast to the clinical team’s impression, the probability of PD remained low throughout, with the main differential during the pre-Parkinsonian prodrome being PSP, CBD or MSA

# **S4. Unique Human Phenotyping Ontology terms**

1. Abnormal autonomic nervous system physiology
2. Abnormal central sensory function
3. Abnormal pyramidal sign
4. Abnormal saccadic eye movements
5. Abnormal social behavior
6. Abnormal sudomotor regulation
7. Abnormality of eye movement
8. Abnormality of vision
9. Acalculia
10. Action myoclonus
11. Action tremor
12. Aggressive behavior
13. Agitation
14. Agraphia
15. Akathisia
16. Akinetic mutism
17. Alien limb phenomenon
18. Allodynia
19. Anarthria
20. Anomic aphasia
21. Anosmia
22. Antecollis
23. Anxiety
24. Apathy
25. Aphasia
26. Apnea
27. Appendicular spasticity
28. Appendular rigidity
29. Apraxia
30. Arm dystonia
31. Asymmetric apraxia
32. Ataxia
33. Auditory hallucinations
34. Autonomic erectile dysfunction
35. Autonomic gastroparaesis
36. Axial dystonia
37. Axial rigidity
38. Babinski sign
39. Back pain
40. Behavioral abnormality
41. Blepharospasm
42. Blurred vision
43. Bowel incontinence/Autonomic dysfunction of the bowel
44. Bradykinesia
45. Bradyphrenia
46. Broad-based gait
47. Bulbar signs
48. Camptocormia
49. Cervical dystonia
50. Chorea
51. Choreoathetosis
52. Clonus
53. Clumsy limb
54. Cognitive impairment
55. Confabulation
56. Confusion
57. Constipation
58. Constructional apraxia
59. Craniofacial dystonia
60. Delayed saccadic initiation
61. Delirium
62. Delusions
63. Depression
64. Diminished ability to concentrate
65. Disinhibition
66. Dopaminergic medication wearing off phenomena
67. Dressing apraxia
68. Dysarthria
69. Dysmetria
70. Dysphagia
71. Dysphonia
72. Dystonia
73. Dystonic antecollis
74. Dystonic laterocollis
75. Dystonic retrocollis
76. Early falls
77. Echolalia
78. Echopraxia
79. Emotional blunting
80. Euphoria
81. Expressive aphasia
82. Eyelid apraxia
83. Facial palsy
84. Falls
85. Finger agnosia
86. Fluctuations in cognitive function
87. Fluctuations in consciousness
88. Focal dystonia
89. Foot dorsiflexor weakness
90. Foot dystonia
91. Freezing of gait
92. Freezing of speech
93. Frontal lobe signs
94. Frontal release signs
95. Frontalis overactivity
96. Functional abnormality of the bladder
97. Gait apraxia
98. Gait ataxia
99. Gait disturbance
100. Glabellar reflex
101. Grasp reflex
102. Hallucinations
103. Hand apraxia
104. Hand dystonia
105. Heat intolerence due to autonomic dysfunction
106. Hemidystonia
107. Hemiparesis
108. Hemiplegia
109. Hemispatial neglect
110. Highly asymmetric Parkinsonism
111. Highly asymmetric parkinsonism
112. Hoarse voice
113. Home care
114. Horizontal nystagmus
115. Horizontal supranuclear gaze palsy
116. Horner syndrome
117. Hyperhidrosis
118. Hyperreflexia
119. Hypersexuality
120. Hyperventilation
121. Hypokinesia
122. Hypometric saccades
123. Hypomimic face
124. Hypophonia
125. Impaired distal vibration sensation
126. Inability to walk
127. Inappropriate gregariousness
128. Inappropriate jocularity
129. Inappropriate laughter
130. Insomnia
131. Inspiratory sighs
132. Intention tremor
133. Intrinsic hand muscle atrophy
134. Irritability
135. Jaw hyperreflexia
136. Joint contracture of the hand/joint contractures involving the joints of the feet
137. Keratoconjunctivitis sicca
138. Kinetic tremor
139. Left-right disorientation
140. Leg dystonia
141. Levodopa induced dyskinesia
142. Limb apraxia
143. Limb ataxia
144. Limb dystonia
145. Limb muscle weakness
146. Limb myoclonus
147. Limb pain
148. Logopenic variant primary progressive aphasia
149. Major Neurocognitive Disorder
150. Major impairment of memory and learning
151. Major impairment of perceptual-motor function
152. Micrographia
153. Mild motor fluctuations
154. Minor Neurocognitive Disorder
155. Minor impairment of complex attention
156. Minor impairment of executive function
157. Minor impairment of language function
158. Minor impairment of memory and learning
159. Minor impairment of perceptual-motor function
160. Motor fluctuations
161. Motor stereotypy
162. Muscle stiffness
163. Mutism
164. Myoclonic tremor
165. Myoclonus
166. Neuroleptic sensitivity
167. Neurological speech impairment
168. Nystagmus
169. Oculomotor apraxia
170. Oromandibular dystonia
171. Oromotor apraxia
172. Orthostatic hypotension
173. Orthostatic syncope
174. Palilalia
175. Paranoia
176. Paraphasia
177. Parkinsonism
178. Parkinsonism with favorable response to dopaminergic medication
179. Parkinsonism with poor response to dopaminergic medication
180. Paroxysmal bursts of laughter
181. Passive behavior
182. Peak dose dyskinesia
183. Peripheral neuropathy
184. Perseveration
185. Personality changes
186. Photophobia
187. Pill rolling tremor
188. Polyphagia
189. Poor speech
190. Postural instability
191. Postural tremor
192. Poverty of speech
193. Procerus sign
194. Pseudobulbar behavioral symptoms
195. Pseudobulbar paralysis
196. Psychomotor retardation
197. Rapid eye movement sleep behaviour disorder
198. Reduced speech
199. Repetitive compulsive behavior
200. Residential care
201. Respiratory failure
202. Resting tremor
203. Restless legs
204. Retrocollis
205. Rigidity
206. Root reflex
207. Self-neglect
208. Sensory axonal neuropathy
209. Sensory neuropathy
210. Severe motor fluctuations
211. Shuffling gait
212. Sialorrhoea
213. Sleep apnea
214. Slow saccadic eye movements
215. Slowed vertical saccades
216. Slurred speech
217. Small fibre neuropathy
218. Small intestinal dysmotility
219. Snout reflex
220. Socially withdrawn
221. Spastic dysarthria
222. Spasticity
223. Speech apraxia
224. Square wave jerks
225. Stimulus sensitive myoclonus
226. Stooped posture
227. Stridor
228. Suck reflex
229. Supranuclear gaze palsy
230. Tachyphemia
231. Task specific tremor
232. Transcortical motor aphasia
233. Tremor
234. Truncal ataxia
235. Urinary catheter for bladder dysfunction
236. Urinary frequency
237. Urinary incontinence
238. Urinary retention
239. Urinary urgency
240. Vertical nystagmus
241. Vertical supranuclear gaze palsy
242. Vertigo
243. Visual hallucinations
244. Visual misperceptions
245. Vivid dreams

# **S5. Supplementary References:**

1. McMurry JA, Köhler S, Washington NL, et al., Navigating the phenotype frontier: the monarch initiative. *Genetics* 2016; **203(4)**:1491–5.

2. Braak H, Braak E. Neuropathological stageing of Alzheimer-related changes. *Acta Neuropathologica* 1991; **82(4)**: 239–259.

3. Thal DR, Rüb U, Orantes M, Braak H. Phases of A beta-deposition in the human brain and its relevance for the development of AD. *Neurology* 2002; **58(12)**:1791–800.

4. Gorgolewski KJ, Auer T, Calhoun VD, et al., The brain imaging data structure, a format for organizing and describing outputs of neuroimaging experiments. *Scientific data* 2016; **3(1)**:1–9.

5. Wan X, Wang W, Liu J, Tong T. Estimating the sample mean and standard deviation from the sample size, median, range and/or interquartile range. *BMC Medical Research Methodology* 2014; **14(1)**: 1–3.

6. Berg D, Postuma RB, Adler CH, et al., MDS research criteria for prodromal Parkinson’s disease. *Movement Disorders : Official Journal of the Movement Disorder Society* 2015; **30(12)**: 1600–11.

7. Allen M, Poggiali D, Whitaker K, et al., Raincloud plots: a multi-platform tool for robust data visualization. *Wellcome Open Research* 2021; **4:63**: 63

8. Ranran Wang (2023). Sankey Diagram. MATLAB Central File Exchange. <https://www.mathworks.com/matlabcentral/fileexchange/75813-sankey-diagram>

9. Cynthia Brewer (<http://colorbrewer.org/>)

10. Schlossmacher MG,2004: Case records of the Massachusetts General Hospital. Weekly clinicopathological exercises. Case 27-2004. A 79-year-old woman with disturbances in gait, cognition, and autonomic function
